# Improved correlation of human Q fever incidence to modelled *C. burnetii* concentrations by means of an atmospheric dispersion model

**DOI:** 10.1186/s12942-015-0003-y

**Published:** 2015-04-01

**Authors:** Jeroen PG van Leuken, Jan van de Kassteele, Ferd J Sauter, Wim van der Hoek, Dick Heederik, Arie H Havelaar, Arno N Swart

**Affiliations:** Institute for Risk Assessment Sciences (IRAS), Faculty of Veterinary Medicine, Utrecht University, P.O. Box 80163, 3508 TD Utrecht, The Netherlands; Centre for Infectious Disease Control (CIb), National Institute for Public Health and the Environment (RIVM), P.O. Box 1, 3720 BA Bilthoven, The Netherlands; Environmental Safety (M&V), National Institute for Public Health and the Environment (RIVM), P.O. Box 1, 3720 BA Bilthoven, The Netherlands; Emerging Pathogens Institute, University of Floriday, Gainesville, Florida USA

**Keywords:** Pathogens, Q fever, Atmospheric dispersion modelling, Airborne, *Coxiella burnetii*, Model comparison, Meteorological

## Abstract

**Background:**

Atmospheric dispersion models (ADMs) may help to assess human exposure to airborne pathogens. However, there is as yet limited quantified evidence that modelled concentrations are indeed associated to observed human incidence.

**Methods:**

We correlated human Q fever (caused by the bacterium *Coxiella burnetii*) incidence data in the Netherlands to modelled concentrations from three spatial exposure models: 1) a NULL model with a uniform concentration distribution, 2) a DISTANCE model with concentrations proportional to the distance between the source and residential addresses of patients, and 3) concentrations modelled by an ADM using three simple emission profiles. We used a generalized linear model to correlate the observed incidences to modelled concentrations and validated it using cross-validation.

**Results:**

ADM concentrations generally correlated the best to the incidence data. The DISTANCE model always performed significantly better than the NULL model. ADM concentrations based on wind speeds exceeding threshold values of 0 and 2 m/s performed better than those based on 4 or 6 m/s. This might indicate additional exposure to bacteria originating from a contaminated environment.

**Conclusions:**

By adding meteorological information the correlation between modelled concentration and observed incidence improved, despite using three simple emission profiles. Although additional information is needed – especially regarding emission data - these results provide a basis for the use of ADMs to predict and to visualize the spread of airborne pathogens during livestock, industry and even bio-terroristic related outbreaks or releases to a surrounding human population.

**Electronic supplementary material:**

The online version of this article (doi:10.1186/s12942-015-0003-y) contains supplementary material, which is available to authorized users.

## Background

Airborne transmission of pathogens in the outdoor environment is characterized by dispersion by the wind (horizontally and vertically). Such pathogens are either isolated or clustered cells or spores, or cells or spores attached to particulate matter or dust [1,2]. Well-known examples of airborne pathogens include:

- The foot-and-mouth disease virus (FMDV) (livestock): major outbreaks have occurred in countries including the UK and France (1981) [3], Italy (1994) [4], The Netherlands (2001) [5], and the UK (2001 and 2007) [6,7].
- *Coxiella burnetii* (livestock), a highly pathogenic agent causing Q fever in humans and animals. Major outbreaks have occurred in countries including Switzerland (1983) with 415 human cases [8], the UK (1989) with 137 human cases [9], France (1998–1999) with 73 human cases [10], Germany (2005) with 331 cases [11], and The Netherlands (2007–2010) with >4,000 human cases [12];
- *Legionella pneumophila* (cooling towers and industrial sources). Major outbreaks have occurred, for example, in France (2003–2004) with 86 human cases including 18 fatalities [13], Norway (2005) with 56 human cases including 10 mortalities [14], and the Netherlands (2006) with 31 human cases [15].
- Avian influenza virus (livestock): outbreaks have occurred world-wide [16];
- *Bacillus anthracis* (‘anthrax’): one described outbreak occurred in the former Soviet Union (1979) [17].

In the case of an early phase of a (future) pathogen outbreak or release – generally related to (animal) industries or to bio-terrorism – it is, from a public and animal health perspective and for economic reasons [18], necessary to require insight in 1) the physical spatial spread of the pathogen, 2) the population at risk, and 3) the concentrations (infectious dose) to which persons and/or animals are exposed.

Traditional epidemiological spatial analysis techniques, such as the attack rate analysis, are however only useful for retrospective analyses and do not incorporate meteorological information [19]. Atmospheric dispersion models (ADMs) – mechanistic models developed to model the spread of particles and gasses spatially and temporarily as a function of meteorological conditions including wind speed and wind direction – may however be instrumental to simulate the spatial spread of pathogens released from a known source. Currently, three types of investigations using ADMs to simulate farm-to-farm, human-to-human, farm-to-human, or industrial-to-human airborne transmission may be distinguished: (1) qualitative investigations, in which airborne spread was modelled visually (e.g., [13,14,17,20]); (2) quantitative investigations, in which modelled concentrations were converted to doses and a quantitative microbial risk assessment was elaborated using dose–response models to calculate infection probabilities (e.g., [21,22]); and (3) the development of emergency preparedness systems and decision-support systems to be used during future outbreaks or releases (e.g., [23-25]). With respect to points 1 and 2, airborne transmission was generally indicated in case modelled concentrations near infected farms or humans exceeded threshold values or if infection probabilities were non-zero.

However, to our knowledge no studies have been published analysing the relationship between reported incidence rates, and concentrations modelled by ADM, using proper quantitative statistical measures. We wish to answer the question: are meteorological models indeed useful to explain observed incidence rates or disease notifications, or could the observed data also be explained by simpler models containing no meteorological information?

Therefore, we aimed at assessing quantitatively whether ADMs improve the correlation between modelled concentrations and observed human disease incidence rates. We used data from the large Q fever outbreaks in the Netherlands [12], and correlated ADM concentration levels to human disease incidence and compared these fits to more simple concentration models that do not contain meteorological information, namely 1) a model with a spatially uniform concentration, and 2) a model with concentration levels proportional to distance from the source.

If the ADM concentration levels correlate better with the human Q fever incidences than the concentration levels of the simple models, we then conclude that ADMs might be useful to predict and visualize the spatial and temporal pathogenic spread in case of an outbreak or release.

## Method

### Data

#### Human case data

**Figure 1:**
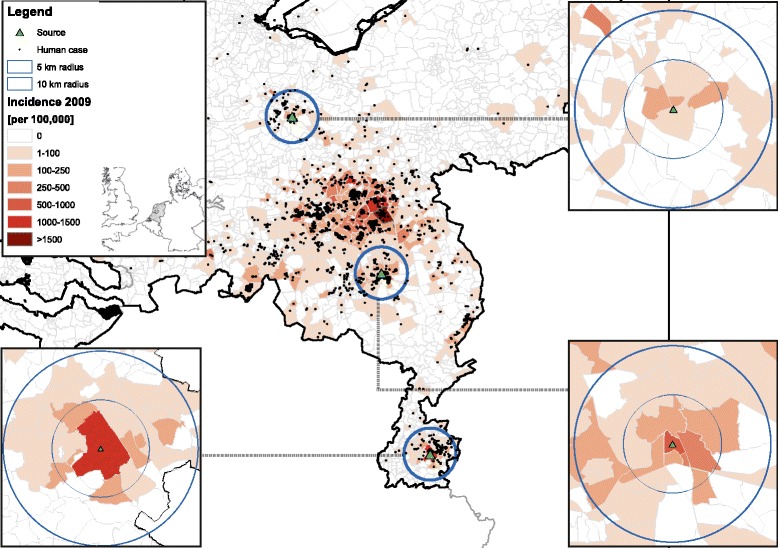
**Q fever incidence map.** Map of the Q fever incidence (per 100,000 inhabitants) in the Netherlands in 2009, and the location of the three selected areas with their main source of exposure.

**Figure 2:**
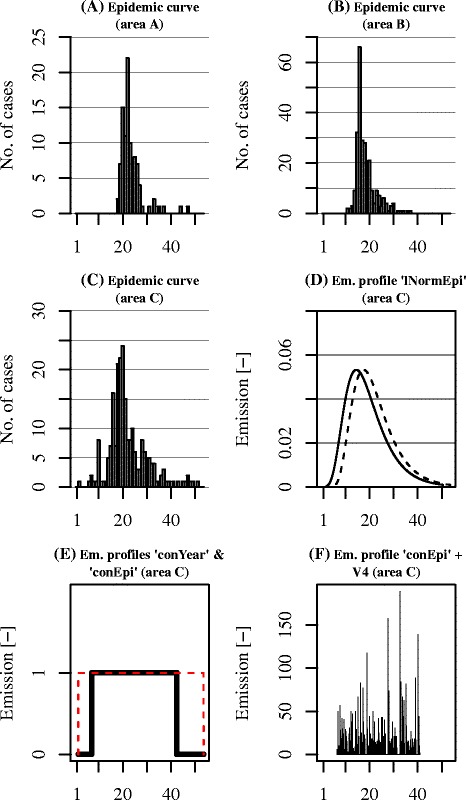
**Epidemic curves and emission profiles. (A/B/C)** Epidemic curves of the three selected areas in number of cases per week; **(D)** lognormal emission profile of area C (*lNormEpi*), both as fit of the epidemic curve (dotted) and shifted 20.7 days back in time (solid); **(E)** steady-state emission profile during 2009 (*conYear*, dotted) and steady-state emission profile during epidemic (*conEpi,* solid) in area C; **(F)** idem as emission profile *conEpi* in subplot E, but with a threshold wind velocity of 4 m/s.

Human case data have been made available by the Municipal Health Services in the Netherlands at the six-digit zip code level (PC6), i.e. street-level. Following [26], we focused on three relatively isolated Q fever outbreak areas where humans experienced exposure to *C. burnetii* from a large dairy goat farm as unique source in 2009. These areas include the Dutch provinces of Utrecht (area A), Noord-Brabant (area B), and Limburg (area C) [19,27] (Figure 1). The epidemic curves per week number in 2009 are shown in Figure 2A, B and C. The specific farms were classified as source in different investigations based on bulk tank milk tests, reported abortion numbers, epidemiological research, and a source detection method [19,26-30].

#### Population density data

Population density data at the PC6-level (reference date 1 January 2010) have been made available by Statistics Netherlands (CBS). The typical maximum distance between a *C. burnetii* infected farm and a case’s home address is 5 – 10 km [19,26]. We therefore selected two arrays of data: PC6’s up to 5000 m from the source, and all PC6’s up to 10 km away (Table 1). Dutch legislation allows using this case information for research purposes if information is not traceable to individual patients. In this case, consent of cases is not required. The case information can however not be made publicly available.

**Table 1:** **Number of cases, number of inhabitants (including cases), incidence per 100,000 inhabitants, and number of zip codes within 5 km and 10 km from the sources in areas A, B, and C**

	**# Cases**		**# Inhabitants**		**Incidence per 100,000**		**# Zip codes**	
	**5 km**	**10 km**	**5 km**	**10 km**	**5 km**	**10 km**	**5 km**	**10 km**
A	66	101	120,090	494,533	55	20	3,025	12,842
B	215	267	75,720	181,195	283	147	1,836	4,526
C	131	220	34,729	228,778	377	96	977	6,095

#### Farm data

Coordinates of the source farms have been made available by the Ministry of Economic Affairs (reference date November 2009).

### Concentration data

#### Simple models

The simple concentration models include:

1. A *NULL* model with a homogeneous concentration in space and time.
2. A *DISTANCE* based model with concentrations proportional to the distance between residential addresses of Q fever patients and the farms.

#### Atmospheric dispersion model

An atmospheric dispersion model (ADM) is a mechanistic model that calculates the physical dispersion of particles and gasses over space and time as a function of emission data and meteorological conditions. We used the *Operational Priority Substances Short Term* model (version 10.3.2), developed by the Netherlands National Institute for Public Health and the Environment (RIVM) (e.g., [31-34]). We considered particulate matter (PM_10_) to be a substitute for *C. burnetii*. The atmospheric dispersion model takes into account both dry and wet deposition of particles.

The OPS model requires hourly-based meteorological data (temperature, relative humidity, wind speed, wind direction, precipitation amounts, precipitation duration, global/solar radiation, and snow cover status) as input for the calculations. These data were retrieved from the Royal Netherlands Meteorological Institute (KNMI) and were determined at the meteorological stations. We spatially interpolated these data to obtain values at farm locations (see Additional file 1: Text S1 and Additional file 2: Figure S16, Additional file 3: Figure S17 and Additional file 4: Figure S18 for a detailed description of the meteorological data preparation). Precipitation data was deduced from precipitation radar images and was available at a 1 km resolution.

The output of the OPS-ST model consisted of hourly averaged PM_10_-concentration matrices (250 m resolution), which we converted to period-specific (see next section) averaged concentration maps. We normalized the concentration values per PC6 to the maximum concentration in the grid (i.e. the concentration at the source).

#### Emission profiles (ADM)

No data were available on the emission strength of *C. burnetii.* Therefore we defined three simple emission profiles (Table 2):

**Table 2:** **Characteristics of the three simple emission profiles as input for the ADM model**

**Name**	**Type**	**Period**	**Comments**
conYear	Constant	[1-Jan-2009; 31-Dec-2009]	
lNormEpi	Lognormal: lagged 20.7 days back in time (mean incubation period)	[1-Jan-2009; 31-Dec-2009]	Area A: μ = 3.1 weeks, σ = 0.20 weeks
			Area B: μ = 2.9 weeks, σ = 0.25 weeks
			Area C: μ = 2.9 weeks, σ = 0.29 weeks
			Calculated with R function MASS∷fitdistr().
conEpi	Constant	Area A: [21-Mar-2009; 31-Jul-2009]	Based on the 2.5% and 97.5% percentile of profile ‘lNormEpi’.
		Area B: [19-Feb-2009; 25-Jun-2009]	
		Area C: [11-Feb-2009; 28-Jun-2009]

A) Emission profile “*conYear*”: steady-state emission strength during the entire period (year 2009) (Figure 2E).
B) Emission profile “*lNormEpi*”: a lognormal emission profile based on the epidemic curve per area, which corresponds well with the lambing season at the farms (Figure 2D).
C) Emission profile “*conEpi*”: constant emission strength starting at the day of the 2.5% percentile and ending at the day of the 97.5% percentile of the lagged profile “*lNormEpi*” (Figure 2E), that is from 27 March to 5 July (area A), 23 February to 8 June (B), and 6 February to 4 October (C).

Although the actual emission profiles will have been much more complex, there is some biological justification for the simple profiles. If one ignores wind direction and meteorological conditions, then a steady-state emission profile is related to a steady-state exposure level. A steady-state exposure seems plausible if goats were shedding bacteria successively during a certain period and/or the farm’s surrounding environment was contaminated as well – the inactivation rate of *C. burnetii* is very low [35] - therefore leading to multiple sources.

A lognormal emission profile could be related to a combination of processes: (1) a (very) short period of high shedding occurred, leading to a normal epidemic curve since the incubation period is distributed normal, or to a lognormal epidemic curve as a result of the normal-distributed incubation period in combination with a contaminated environment; (2) the shedding rate was time-dependent and followed a (log)normal curve, potentially leading to a lognormal epidemic curve if a contaminated environment is considered as well.

In addition we considered four threshold wind speeds for emission of *C. burnetii*, namely 0, 2, 4 and 6 m^.^s^−1^ (profiles *V0*, *V2*, *V4*, and *V6* respectively). In the case where the hourly wind speed at the farm was lower than the threshold value, we assumed that bacteria would accumulate in the stable and would be released during the next hour that the wind speed threshold was exceeded (Figure 2F). The reason for this choice is that stables of large dairy goat farms are very open to the outdoor environment; thus, pathogens deposited on stable floors and surfaces can easily be aerosolized by strong enough winds, and then be dispersed to the farm’s surrounding environment.

### Statistical analysis

#### Incidence versus concentration

The dose–response relationship for infectious micro-organisms like *C. burnetii* is given by (e.g., [36]):

1 $$ {p}_i=1- \exp \left(-\kappa {\lambda}_i\right) $$

with *p*_*i*_ being the probability of infection at PC6 *i*, *κ* being the single-hit probability of initiating infection, and *λ*_i_ being the dose at PC6 *i* [number of pathogens]. Since the observed overall incidence of Q fever during the Dutch epidemic is relatively small (Table 1), we can assume that the doses *λ* were relatively small too. Since exp(*λ*) ≅ 1 + *λ* for small values of *λ*, equation [1] approaches a linear equation:

2 $$ {p}_i\cong \kappa \cdot {\lambda}_i $$

For each PC6 *i* we determined the number of cases *k*_i_ and inhabitants *n*_i_. Assuming that the probability of infection *p* is equal to the incidence *I*, and that the log-dose *λ* is proportional to the log-concentration, one could test which concentration model (NULL, DISTANCE, or ADM with the emission and wind speed threshold configurations) gives the best fit to the incidence by means of a Poisson generalized linear model (R version 3.0.3):

*k*_*i*_ ~ Poisson(*μ*_*i*_)

3 $$ \log \left(\overrightarrow{\mu}\right)= \log \left(\overrightarrow{n}\right)+{\beta}_0+{\beta}_1\cdot \log \left[f\left(\overrightarrow{x}\right)\right] $$

where *μ* is the expected outcome, *β*_0_ and *β*_1_ are the intercept and slope of the log-linear fit, and *f(x)* is the concentration function. In order to fulfil the linear conditions of equation [2], the slope should approximate 1 (i.e. *β*_1_ ≈ 1):

4 $$ \exp \left[ \log \left(\overrightarrow{\mu}\right)\right]= \exp \left[ \log \left(\overrightarrow{n}\right)+{\beta}_0+{\beta}_1 \log \left[f\left(\overrightarrow{x}\right)\right]\right] $$

which equals

5 $$ \frac{\overrightarrow{\mu}}{\overrightarrow{n}}= \exp \left[{\beta}_0\right]\cdot f{(x)}^{\beta_1} $$

and thus exp[*β*_0_] ~ *κ*.

For the NULL model, we defined *f(x)* = 1, for the DISTANCE model $f(x)={\overrightarrow{r}}^{-2}$, and for the ADM-models *f(x)* is a function of a large set of meteorological equations.

#### Model comparison

To compare the performance of the NULL, DISTANCE and ADM models we applied a cross validation test [37]. That is, for each of these models we randomly selected 2/3 of the number of PC6’s (training data) and estimated the intercept (*β*_0_) and the slope (*β*_1_) of equation [3]. Subsequently, we applied that linear model to the remaining 1/3 of the data (test data), predicted their outcome, and calculated the residual deviances (*δ*_c_) for each cross validation test *c*. Finally, we calculated the total residual deviance (*d*_c_), being the sum of the residual deviances. We repeated this cross validation test 10,000 times, and calculated the mean total residual deviance *D* per concentration model:

6 $$ D=\frac{1}{v}\cdot {\displaystyle \sum_p}{d}_p=\frac{1}{v}\cdot {\displaystyle \sum_c}{\left({\displaystyle \sum_q}{\delta}_q\right)}_c $$

with *v* = 10,000 and *c* = 1…v.

Finally, we compared the *D-*values of the different concentration models by means of a two-sample t-test with 5% significance.

## Results

### Mean total residual deviance (D)

**Figure 3:**
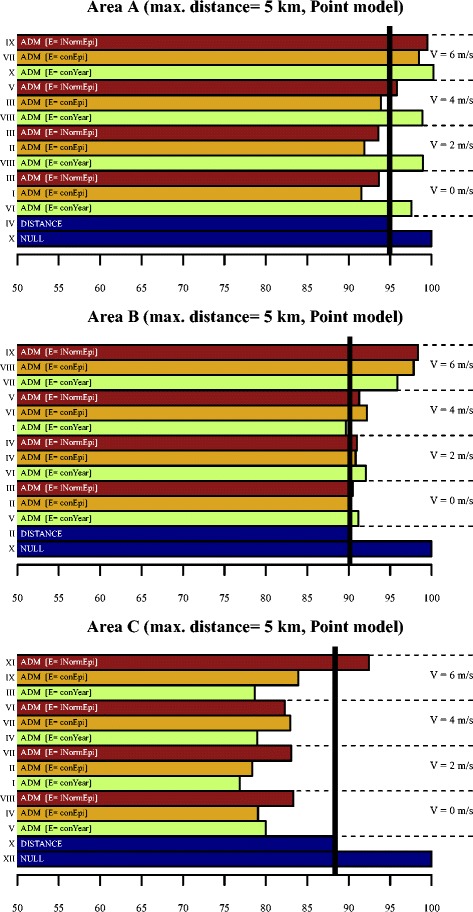
**D-values (5 km).** *D*-values of the NULL, DISTANCE and ADM-models, relative to the *D*-value of the NULL model, based on all PC6’s within 5 km of the source. The vertical black line represents the *D*-value of the DISTANCE model. The Roman numerals refer to groups of models with a significantly equal *D* value (I = significantly lowest *D*-value; II = *D* significantly lower than those of I, etc.).

**Figure 4:**
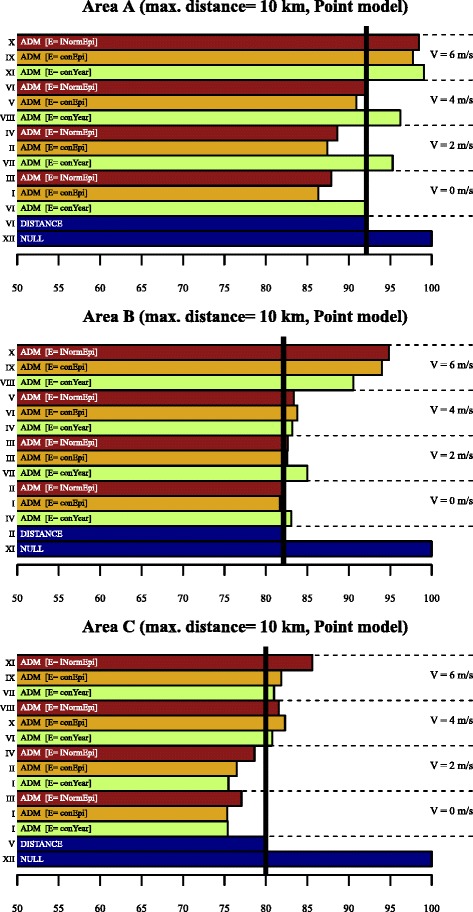
**D-values (10 km).** Idem as Figure 3, but based on all PC6’s within 10 km of the source.

Figure 3 and Table 3 show that in *area A* the ADM’s with profiles *conEpi-V0* and *conEpi-V2* have the lowest *D* (±7.8% lower than the NULL model). The DISTANCE model has a 4.5% lower *D* than the NULL model. The ADMs with an annual constant emission profile (*conYear*) always performed worse than the DISTANCE model; this corresponds well with the observed short duration of the epidemic in this area (Figure 2A). The ADMs with profile *V6* performed approximately equal to the NULL model. Increase of the selection radius to 10 km (Figure 4, Table 4) did not lead to major changes, although the indexed *D*’s are lower. Note that *D for* the *conEpi*-*V0* model is significantly lower than that of the *conEpi-V2 model*.

**Table 3:** ***Five km results*** **with mean total residual deviances (** ***D*** **) and each model’s ranked position**

**Area**	**Model**	**Emission**	***V***	**β**_**0**_ **(intercept)**			**β**_**1**_ **(slope)**			***D***	**Position**
				***μ***	**95% CI**	***p*** ^*^	***μ***	**95% CI**	***p*** ^*^		
A	ADM	conEpi	0	−2.48	[−4.03;-0.94]	4.5E-03	0.99	[0.67;1.31]	9.9E-06	150.9	I
A	ADM	conEpi	2	−2.64	[−4.22;-1.07]	2.6E-03	0.90	[0.59;1.20]	1.8E-05	151.3	I
A	ADM	lNormEpi	0	−2.98	[−4.58;-1.37]	6.5E-04	0.91	[0.58;1.24]	1.0E-04	154.0	II
A	ADM	lNormEpi	2	−3.16	[−4.78;-1.54]	3.5E-04	0.81	[0.50;1.12]	1.6E-04	154.5	II
A	ADM	conEpi	4	−3.74	[−5.30;-2.17]	2.4E-05	0.58	[0.33;0.83]	2.0E-04	155.1	III
A	DISTANCE	-	-	−2.33	[−4.30;-0.35]	*5.4E-02*	0.59	[0.36;0.81]	8.8E-04	156.5	IV
A	ADM	lNormEpi	4	−4.37	[−5.93;-2.82]	6.2E-07	0.49	[0.24;0.74]	1.9E-03	157.8	V
A	ADM	conYear	0	−4.45	[−6.24;-2.66]	2.4E-06	0.60	[0.24;0.96]	1.2E-02	161.0	VI
A	ADM	conYear	4	−5.54	[−7.29;-3.79]	8.3E-09	0.31	[0.03;0.59]	*7.6E-02*	162.8	VII
A	ADM	conEpi	6	−5.74	[−7.19;-4.29]	3.6E-09	0.27	[0.05;0.49]	*5.3E-02*	162.9	VII
A	ADM	conYear	2	−5.33	[−7.28;-3.39]	2.4E-07	0.39	[0.04;0.75]	*8.5E-02*	163.8	VIII
A	NULL	-	-	−7.51	[−7.80;-7.21]	0	-	-	-	163.9	VIII
A	ADM	lNormEpi	6	−6.19	[−7.67;-4.70]	1.4E-11	0.19	[−0.02;0.40]	*1.5E-01*	164.0	VIII
A	ADM	conYear	6	−6.75	[−8.44;-5.06]	4.6E-12	0.11	[−0.13;0.36]	*4.1E-01*	165.1	IX
B	ADM	conYear	4	−1.24	[−2.25;-0.24]	4.7E-02	0.74	[0.57;0.90]	2.6E-11	264.1	I
B	DISTANCE	-	-	−2.00	[−2.79;-1.20]	2.1E-03	0.72	[0.57;0.88]	2.6E-12	265.0	II
B	ADM	conEpi	0	−1.89	[−2.72;-1.05]	4.8E-03	0.85	[0.67;1.04]	3.1E-12	265.5	II
B	ADM	lNormEpi	0	−2.01	[−2.83;-1.19]	3.0E-03	0.84	[0.65;1.02]	5.5E-12	266.1	III
B	ADM	conEpi	2	−1.88	[−2.78;-0.98]	3.9E-03	0.78	[0.60;0.96]	5.7E-11	267.0	IV
B	ADM	lNormEpi	2	−1.94	[−2.82;-1.05]	4.2E-03	0.77	[0.59;0.95]	3.7E-10	267.3	IV
B	ADM	conYear	0	−1.62	[−2.51;-0.73]	2.9E-02	0.84	[0.66;1.02]	2.9E-11	268.4	V
B	ADM	lNormEpi	4	−2.14	[−3.09;-1.18]	3.7E-04	0.64	[0.47;0.81]	5.9E-10	268.4	V
B	ADM	conEpi	4	−2.36	[−3.31;-1.42]	6.0E-05	0.59	[0.43;0.76]	5.9E-09	270.6	VI
B	ADM	conYear	2	−1.56	[−2.51;-0.61]	4.4E-02	0.77	[0.59;0.94]	7.1E-11	270.8	VI
B	ADM	conYear	6	−3.01	[−4.05;-1.98]	7.6E-06	0.40	[0.25;0.55]	2.8E-05	281.9	VII
B	ADM	conEpi	6	−4.52	[−5.22;-3.82]	5.4E-23	0.18	[0.09;0.28]	1.7E-03	288.2	VIII
B	ADM	lNormEpi	6	−4.52	[−5.28;-3.75]	1.1E-22	0.18	[0.08;0.28]	4.5E-03	289.2	IX
B	NULL	-	-	−5.86	[−6.03;-5.70]	0	-	-	-	294.1	X
C	ADM	conYear	2	1.17	[−0.01;2.35]	*0.11*	1.27	[1.03;1.51]	1.7E-15	156.2	I
C	ADM	conEpi	2	0.99	[−0.24;2.21]	*0.18*	1.22	[0.97;1.46]	1.7E-15	159.8	II
C	ADM	conYear	6	−0.25	[−1.36;0.86]	*0.54*	0.86	[0.66;1.05]	6.6E-13	160.0	II
C	ADM	conYear	4	0.00	[−1.19;1.19]	*0.61*	0.94	[0.73;1.16]	1.4E-12	160.4	III
C	ADM	conEpi	0	1.21	[0.17;2.26]	*0.10*	1.35	[1.13;1.57]	3.9E-19	161.0	III
C	ADM	conYear	0	1.13	[0.07;2.18]	*0.15*	1.34	[1.11;1.56]	4.7E-18	162.8	IV
C	ADM	lNormEpi	4	0.20	[−1.15;1.55]	*0.58*	0.97	[0.73;1.21]	2.7E-11	167.1	V
C	ADM	conEpi	4	−0.06	[−1.42;1.29]	*0.57*	0.91	[0.67;1.15]	1.1E-10	168.6	VI
C	ADM	lNormEpi	0	0.97	[−0.11;2.05]	*0.22*	1.31	[1.08;1.53]	2.5E-17	169.0	VI
C	ADM	lNormEpi	2	0.55	[−0.74;1.84]	*0.45*	1.13	[0.88;1.39]	2.7E-13	169.0	VI
C	ADM	conEpi	6	−0.52	[−1.72;0.69]	*0.42*	0.78	[0.58;0.98]	1.8E-10	170.5	VII
C	DISTANCE	-		−0.28	[−1.39;0.82]	*0.39*	0.98	[0.77;1.20]	1.2E-11	180.3	VIII
C	ADM	lNormEpi	6	−1.81	[−3.08;-0.54]	0.02	0.57	[0.37;0.76]	1.7E-06	187.9	IX
C	NULL	-		−5.58	[−5.79;-5.37]	0.00	-		-	203.8	X

*Italic *p*-values for *β*_0_ and *β*_1_ indicate *p* ≥ 0.05.

**Table 4:** ***Ten km results*** **with mean total residual deviances (** ***D*** **) and each model’s ranked position**

**Area**	**Model**	**Emission**	***V***	**β**_**0**_			**β**_**1**_			***D***	**Position**
				***μ***	**95% CI**	***p*** ^*^	***μ***	**95% CI**	***p*** ^*^		
A	ADM	conEpi	0	−1.97	[−3.04;-0.89]	2.0E-03	1.11	[0.91;1.30]	2.0E-14	273.4	I
A	ADM	conEpi	2	−2.04	[−3.15;-0.93]	1.5E-03	1.03	[0.84;1.22]	3.3E-14	276.8	II
A	ADM	lNormEpi	0	−2.27	[−3.36;-1.18]	2.8E-04	1.07	[0.87;1.28]	9.7E-14	278.2	III
A	ADM	lNormEpi	2	−2.32	[−3.45;-1.20]	2.1E-04	1.00	[0.80;1.19]	8.5E-14	280.1	IV
A	ADM	conEpi	4	−2.77	[−4.01;-1.52]	7.0E-05	0.79	[0.60;0.97]	1.3E-10	288.0	V
A	DISTANCE	-	-	−1.18	[−2.48;0.12]	*2.2E-01*	0.75	[0.61;0.89]	1.1E-11	291.6	VI
A	ADM	conYear	0	−3.19	[−4.37;-2.01]	4.7E-07	0.91	[0.69;1.12]	5.3E-10	292.0	VI
A	ADM	lNormEpi	4	−3.33	[−4.58;-2.09]	1.6E-06	0.72	[0.53;0.90]	4.9E-09	292.3	VI
A	ADM	conYear	2	−3.97	[−5.30;-2.63]	3.2E-08	0.72	[0.50;0.94]	1.3E-06	301.8	VII
A	ADM	conYear	4	−4.44	[−5.83;-3.06]	3.9E-09	0.57	[0.37;0.77]	3.0E-05	304.6	VIII
A	ADM	conEpi	6	−5.45	[−6.81;-4.09]	2.3E-10	0.41	[0.22;0.60]	1.0E-03	309.1	IX
A	ADM	lNormEpi	6	−5.90	[−7.28;-4.52]	1.8E-12	0.34	[0.15;0.52]	4.5E-03	312.5	X
A	ADM	conYear	6	−6.02	[−7.51;-4.54]	3.5E-13	0.33	[0.13;0.52]	1.4E-02	313.5	X
A	NULL	-	-	−8.50	[−8.74;-8.26]	0	-	-	-	316.2	XI
B	ADM	conEpi	0	−1.48	[−2.10;-0.86]	3.1E-03	0.96	[0.83;1.08]	1.4E-32	391.7	I
B	DISTANCE	-	-	−1.63	[−2.23;-1.04]	8.1E-04	0.81	[0.70;0.91]	8.0E-33	393.1	II
B	ADM	lNormEpi	0	−1.57	[−2.18;-0.97]	2.4E-03	0.95	[0.82;1.07]	9.4E-34	393.5	II
B	ADM	conEpi	2	−1.29	[−1.95;-0.64]	8.7E-03	0.92	[0.79;1.04]	4.5E-30	395.1	III
B	ADM	lNormEpi	2	−1.34	[−1.99;-0.69]	9.3E-03	0.91	[0.79;1.03]	2.2E-31	395.5	III
B	ADM	conYear	4	−0.86	[−1.63;-0.09]	*7.4E-02*	0.82	[0.70;0.93]	1.7E-27	397.4	IV
B	ADM	conYear	0	−1.13	[−1.78;-0.47]	4.3E-02	0.96	[0.83;1.08]	1.5E-32	398.0	IV
B	ADM	lNormEpi	4	−1.55	[−2.26;-0.84]	6.3E-04	0.77	[0.65;0.88]	2.9E-27	399.3	V
B	ADM	conEpi	4	−1.62	[−2.33;-0.91]	2.6E-04	0.75	[0.63;0.87]	3.7E-27	401.6	VI
B	ADM	conYear	2	−0.95	[−1.65;-0.24]	*9.7E-02*	0.91	[0.79;1.03]	1.2E-28	406.7	VII
B	ADM	conYear	6	−2.40	[−3.20;-1.59]	2.0E-05	0.53	[0.42;0.64]	1.0E-14	433.2	VIII
B	ADM	conEpi	6	−3.74	[−4.43;-3.04]	3.2E-19	0.36	[0.26;0.45]	6.5E-10	449.3	IX
B	ADM	lNormEpi	6	−3.74	[−4.49;-2.99]	3.4E-15	0.35	[0.25;0.44]	1.4E-08	455.0	X
B	NULL	-	-	−6.52	[−6.67;-6.37]	0	-	-	-	479.0	XI
C	ADM	conEpi	0	1.36	[0.61;2.11]	8.5E-03	1.39	[1.26;1.53]	8.7E-49	386.5	I
C	ADM	conYear	2	1.63	[0.80;2.46]	3.0E-03	1.39	[1.25;1.54]	2.0E-48	387.1	I
C	ADM	conYear	0	1.35	[0.61;2.10]	9.4E-03	1.40	[1.26;1.54]	6.6E-49	388.0	II
C	ADM	conEpi	2	1.54	[0.68;2.40]	6.5E-03	1.36	[1.21;1.51]	1.1E-42	392.6	III
C	ADM	lNormEpi	0	1.23	[0.47;1.98]	1.8E-02	1.38	[1.24;1.52]	8.7E-45	395.2	IV
C	ADM	lNormEpi	2	1.34	[0.46;2.23]	1.9E-02	1.33	[1.18;1.48]	1.1E-39	403.3	V
C	DISTANCE	-	-	0.36	[−0.35;1.07]	*4.4E-01*	1.13	[1.01;1.25]	9.1E-45	411.0	VI
C	ADM	conYear	4	0.36	[−0.58;1.29]	*4.3E-01*	1.08	[0.93;1.23]	1.3E-24	415.1	VII
C	ADM	conYear	6	−0.17	[−1.08;0.74]	*5.2E-01*	0.95	[0.81;1.09]	1.8E-23	416.4	VIII
C	ADM	lNormEpi	4	0.71	[−0.29;1.71]	*2.7E-01*	1.12	[0.96;1.28]	3.8E-21	418.6	IX
C	ADM	conEpi	6	−0.05	[−1.01;0.91]	*5.6E-01*	0.93	[0.79;1.07]	7.3E-25	420.9	X
C	ADM	conEpi	4	0.43	[−0.59;1.46]	*4.1E-01*	1.07	[0.91;1.23]	7.2E-23	422.6	XI
C	ADM	lNormEpi	6	−0.68	[−1.66;0.30]	*2.6E-01*	0.83	[0.69;0.97]	1.4E-21	439.3	XII
C	NULL	-	-	−6.95	[−7.11;-6.79]	0	-	-	-	512.8	XIII

*Italic *p*-values for *β*_0_ and *β*_1_ indicate *p* ≥ 0.05.

In *area B*, the results differ considerably compared to area A. The *D*-values of all models, except for the ADM-models with *V6*, are approximately 10% lower than that of the NULL model. The differences in *D* between the DISTANCE model and the ADM’s with *V0*, *V2* and *V4* are small, but the model with *conYear-V4* still performs significantly better than the DISTANCE model (p < 0.05).

For the 10 km selection radius (Figure 4), the *D*-values are ± 17% lower than that of the NULL model (ADM *V6*-models not included). In this case, *conEpi-V0* gives the best performance, but the differences in *D* with respect to the DISTANCE and ADMs with *V0*, *V2* and *V4* remain small.

In *area C* the difference in *D* between the DISTANCE and the NULL model is 11.5%, and all ADM’s (except for *lNormEpi-V6*) performed better with a *D* of ± 16-23% lower than that of the NULL model. The best performance is given by *conYear-V2* (seeming to correspond with the longer duration of the epidemic as depicted in Figure 2C). Note that in general the ADM’s with *lNormEpi* have the highest *D*, and that the ADMs with *conYear* all result in relatively low *D*-values, even with V4 and V6.

For the 10 km selection radius (Figure 4) the *D*-values of all models improves compared to the NULL model, but all ADM’s with *V4* and *V6* have a significantly higher *D* than the DISTANCE model. The ADM’s with profiles *conEpi-V0* and *conYear-V2* have the lowest *D*-values.

Additional file 5: Figure S1, Additional file 6: Figure S2 and Additional file 7: Figure S3 show the ADM concentration plots for all emission curves and threshold wind velocities of areas A, B and C. Additional file 8: Figure S4, Additional file 9: Figure S5, Additional file 10: Figure S6, Additional file 11: Figure S7, Additional file 12: Figure S8 and Additional file 13: Figure S9 show the predicted versus observed incidence rates per PC6 of areas A, B and C as a function of the selection radii of 5 and 10 km. Additional file 14: Figure S10, Additional file 15: Figure S11, Additional file 16: Figure S12, Additional file 17: Figure S13, Additional file 18: Figure S14 and Additional file 19: Figure S15 show the geographical plots of the observed and predicted incidence rates.

### Validation of the dose–response linearity

From equation [2] and [5] we inferred that ideally the slope of the log-linear fits (${\beta}_1$) would approximate 1 if the overall doses were relatively low. In area A, this boundary condition is met for the four best models (considering the 95% confidence interval) (Tables 3 and 4). In areas B and C, the condition is (almost) met for the majority of the models. These results support a linear dose response relation.

## Discussion

In the current study, we correlated observed Q fever incidence numbers to modelled averaged concentrations of *C. burnetii* from two simple concentration models (NULL and DISTANCE) and of an atmospheric dispersion model with varying emission profiles and threshold wind speed. If all cases were uniformly distributed over the outbreak area, the model with the spatially homogeneous concentration (NULL model) should have had the best fit.

Instead, the DISTANCE model always performed better than the NULL model, which is possibly due to clustering of cases around the infected farms (a similar pattern was observed in previous study [26]). In addition, the observed incidence numbers correlated significantly better to the concentrations of some ADM models in all areas (but with small differences compared to the DISTANCE model in one of the three areas), indicating that meteorological conditions might have played a role during the Dutch Q fever epidemic.

The best fitting ADM models all had a slope in the log-linear fit (*β*_1_) approximately equal to 1 or with the same order of magnitude. This might be an indication for relatively low doses, as is supported by *C. burnetii* measurements that were performed in 2009 [38].

In this study we applied a threshold wind speed for emission for two reasons. First, turbulent movements of the air are required to aerosolize the bacteria deposited on stable surfaces by dairy goats. Secondly, introduction of a threshold wind speed caused the annually averaged concentrations to be direction dependent (Figures S1-S3).

We conclude that, in general, the ADMs with a threshold wind speed of 0 or 2 m/s performed best. However, for aerosolization higher threshold wind speeds are required generally [39], especially in case of rough terrain such as a farm environment. We think two physical explanations exist for the relatively better performance of V0 and V2 profiles. Firstly, a sufficient amount of bacteria may have been aerosolized in the stable not only by the wind, but also by physical activity within the stable (e.g., feeding operations and movements of goats). Secondly, the surrounding environment of the goat stables may have been contaminated during the epidemic – given the high persistence of *C. burnetii* in the environment [35] – and thus a larger surface source may have developed. Thus, cases could have been infected from a wider range of wind directions.

Additional file 8: Figure S4, Additional file 9: Figure S5, Additional file 10: Figure S6, Additional file 11: Figure S7, Additional file 12: Figure S8 and Additional file 13: Figure S9 show the scatterplots of the predicted versus observed incidence rates in the three areas; Additional file 14: Figure S10, Additional file 15: Figure S11, Additional file 16: Figure S12, Additional file 17: Figure S13, Additional file 18: Figure S14, Additional file 19: Figure S15 and Additional file 2: Figure S16 show the geographical prediction plots. These plots make clear that the statistical Q fever incidence prediction (in a statistical sense) should be improved further. In general, the data are rather scattered and not close to the 1×1 line. We think several causes may explain the moderate predictability:

1. Lack of actual emission data, as a result of which actual concentrations might have deviated significantly from modelled concentrations.
2. Timing of concentration values (linked to point 1). Since time-dependent emission curves were unknown, we correlated observed incidence rates to *cumulative* concentration values. However, in reality, cases might have been infected after exposure to a particular dose or particular cumulative dose.
3. Presence of a contaminated environment. The total Q fever epidemic in the Netherlands lasted four years with seasonal outbreaks. Although we specifically focused on 2009, infections also occurred in other years in the selected areas. Results from both bulk tank milk sampling [28] and goat vaginal swab sampling [38,40,41] indicated that the source farms had already been positive in 2008. Indeed, the farm in area B had already caused a human outbreak in 2008 [19]. This may have resulted in an already contaminated environment prior to 2009 [42,43], favoured by the relatively low decay rate of *C. burnetii* [35]. A combination of emission from infected farms as well as emission from contaminated surrounding environments could have resulted in the clear absence of a (wind) direction dependent incidence pattern in area B.
4. Complex human mobility patterns: the health outcome of exposure to an infectious agent is generally dependent on the type and virulence of the pathogen, its concentration (or infectious dose), the exposure (both frequency and duration) and the immune status and general health status of the susceptible host. In that perspective, actual exposure is more difficult to assess in the case of humans than in the case of animals, since humans are very mobile. Although Dutch people spend approximately 70% of their time at home [44], it is very well possible that cases might have been infected on other locations than their own PC6, or non-cases might have ‘missed’ out on days with high exposure on their PC6. In this study we did not have information on case activity patterns, although this might be important for a better risk analysis as a very small number of bacteria are able to cause infection [45,46]. Nevertheless, since the ADM correlation results were best for area C and since the results in our previous distance-based study showed much more contrast in area C compared to the other areas [26], we think that either the fraction of infected persons in area C that were indeed infected *within their PC6* was higher than in the other areas, or other sources of *C. burnetii* were not present in this area (or a combination of both).
5. Spatially heterogeneous awareness of Q fever among the population, resulting in a bias in the observed incidence.
6. Protective immunity from childhood [30] or immunity caused by infections in 2007 or 2008. A recent study confirmed that in humans with acute Q fever the level of antibodies remains high for several years [47].
7. Infection by other sources, either by a (small) unknown source in the areas themselves, or by a source in another part of the country.

Nevertheless, we showed that concentrations based on meteorological conditions correlated better to observed incidences than the NULL and DISTANCE based models, despite the fact that (1) actual emission data was lacking (thus simple emission profiles were useful), (2) the total exposure time was quite long, and (3) probable transmission by a contaminated environment could have influenced the observations.

We recommend repeating this study using similar data sets, and to repeat it for outbreaks or releases with airborne transmission during a relatively short period. That way, airborne pathogenic transmission to humans could be separated easier from transmission from a contaminated surrounding environment. In addition, it would be necessary to determine realistic emission strengths for *C. burnetii* to calculate exposure levels and infection probabilities using dose–response models [46].

To our knowledge this is the first study that attempts to quantify applicability of an atmospheric dispersion model for a pathogen outbreak considering human infections during an outbreak. Our results indicated that ADMs yield some promising results and that they can be used for livestock related outbreaks although more extensive validation work is needed, under different circumstances. This may make ADMs to serve as tools for environmental planning purposes to visualize and predict the spread of microbes from farms and industries to surrounding human populations.

## Additional files

Additional file 1: Text S1. Meteorological data processing. Additional file 2: Figure S16. Interpolation maps (1). Measurements and interpolated data of global radiation, relative humidity, temperature and snow cover status for randomly selected hours in 2009. Additional file 3: Figure S17. Interpolation maps (2). Measured and interpolated wind speed and wind direction for a randomly selected hour in 2009. Additional file 4: Figure S18. Precipitation radar image. A semi-random selected precipitation radar image with the converted precipitation intensity at several locations in the Netherlands. Additional file 5: Figure S1. Concentration maps (area A). Log-transformed ADM concentration maps (relative to the maximum concentration in the grid) with emission profiles *conYear*, *conEpi*, and *lNormEpi,* and threshold wind speed profiles V0, V2, V4 and V6 (area A). Additional file 6: Figure S2. Concentration maps (area B). Log-transformed ADM concentration maps (relative to the maximum concentration in the grid) with emission profiles *conYear*, *conEpi*, and *lNormEpi,* and threshold wind speed profiles V0, V2, V4 and V6 (area B). Additional file 7: Figure S3. Concentration maps (area C). Log-transformed ADM concentration maps (relative to the maximum concentration in the grid) with emission profiles *conYear*, *conEpi*, and *lNormEpi,* and threshold wind speed profiles V0, V2, V4 and V6 (area C). Additional file 8: Figure S4. Predicted versus observed incidence rates (area A, 5 km). Area A, selection radius 5 km: Predicted (y) versus observed (x) incidence rates per PC6 for the NULL, DISTANCE and ADM models. The solid line displays the 1×1 curve. PC6’s with no observed cases are not included. Additional file 9: Figure S5. Predicted versus observed incidence rates (area B, 5 km). Area B, selection radius 5 km: Predicted (y) versus observed (x) incidence rates per PC6 for the NULL, DISTANCE and ADM models. The solid line displays the 1×1 curve. PC6’s with no observed cases are not included. Additional file 10: Figure S6. Predicted versus observed incidence rates (area C, 5 km). Area C, selection radius 5 km: Predicted (y) versus observed (x) incidence rates per PC6 for the NULL, DISTANCE and ADM models. The solid line displays the 1×1 curve. PC6’s with no observed cases are not included. Additional file 11: Figure S7. Predicted versus observed incidence rates (area A, 10 km). Area A, selection radius 10 km: Predicted (y) versus observed (x) incidence rates per PC6 for the NULL, DISTANCE and ADM models. The solid line displays the 1×1 curve. PC6’s with no observed cases are not included. Additional file 12: Figure S8. Predicted versus observed incidence rates (area B, 10 km). Area B, selection radius 10 km: Predicted (y) versus observed (x) incidence rates per PC6 for the NULL, DISTANCE and ADM models. The solid line displays the 1×1 curve. PC6’s with no observed cases are not included. Additional file 13: Figure S9. Predicted versus observed incidence rates (area C, 10 km). Area C, selection radius 10 km: Predicted (y) versus observed (x) incidence rates per PC6 for the NULL, DISTANCE and ADM models. The solid line displays the 1×1 curve. PC6’s with no observed cases are not included. Additional file 14: Figure S10. Geographical observed and predicted incidence map (area A, 5 km). Area A, selection radius 5 km: Geographical observed and predicted incidence rates per 100,000 inhabitants aggregated to a raster at the 250 m level (log_10_-scale). Grey pixels represent incidence rates of 0. Additional file 15: Figure S11. Geographical observed and predicted incidence map (area B, 5 km). Area B, selection radius 5 km: Geographical observed and predicted incidence rates per 100,000 inhabitants aggregated to a raster at the 250 m level (log_10_-scale). Grey pixels represent incidence rates of 0. Additional file 16: Figure S12. Geographical observed and predicted incidence map (area C, 5 km). Area C, selection radius 5 km: Geographical observed and predicted incidence rates per 100,000 inhabitants aggregated to a raster at the 250 m level (log_10_-scale). Grey pixels represent incidence rates of 0. Additional file 17: Figure S13. Geographical observed and predicted incidence map (area A, 10 km). Area A, selection radius 10 km: Geographical observed and predicted incidence rates per 100,000 inhabitants aggregated to a raster at the 250 m level (log_10_-scale). Grey pixels represent incidence rates of 0. Additional file 18: Figure S14. Geographical observed and predicted incidence map (area B, 10 km). Area B, selection radius 10 km: Geographical observed and predicted incidence rates per 100,000 inhabitants aggregated to a raster at the 250 m level (log_10_-scale). Grey pixels represent incidence rates of 0. Additional file 19: Figure S15. Geographical observed and predicted incidence map (area C, 10 km). Area C, selection radius 10 km: Geographical observed and predicted incidence rates per 100,000 inhabitants aggregated to a raster at the 250 m level (log_10_-scale). Grey pixels represent incidence rates of 0.
