# Supplementary material for: Improved correlation of human Q fever incidence to modelled C. burnetii concentrations by means of an atmospheric dispersion model
Source: Int J Health Geogr. 2015 Apr 1;14:14. doi: 10.1186/s12942-015-0003-y (PMC4440286; doi:10.1186/s12942-015-0003-y)
Supplement: Additional file 1: Text S1. — Meteorological data processing. [file 12942_2015_3_MOESM1_ESM.docx]

**Supplementary Text S1 – Meteorological data processing**

*1. Global radiation, temperature, relative humidity, and snow cover*

Hourly global radiation, temperature, relative humidity, and snow cover data were downloaded from the database of the Royal Netherlands Meteorological Institute [1, 2]. These data, available for 34 measurement sites were spatially interpolated to get local values at the positive farms. We used various interpolation techniques for the four parameters, based on two KNMI reports [3, 4]. These reports however do not supply values of specific interpolation parameters. Therefore we determined plausible values by performing sensitivity analyses (non-formal) in combination with visual inspection.

- Global radiation (*Q*): interpolated with the Thin Plate Splines (TPS) method (R functions *fields::tps()* and *stats::predict()*), with *λ* = 4x10^-5^.

- Relative humidity (*RH*): ordinary kriging (OK) was used by [4] to interpolate annually averaged relative humidity. However, unrealistic results arose by applying this technique to hourly averaged relative humidity. Therefore, we applied the Inverse Distance Weighted (IDW) method (R function *krige::idw( block= 50000, maxdist= 120000, idp= 5)*).

- Temperature (*T*): interpolated with the IDW method (same configurations as for relative humidity).

- Snow cover status (*S*): interpolated with the OK method (R function *automap::autoKrige( model= ‘Exp’ )*. We assumed that interpolated snow cover values of ≥ 0.5 corresponded to complete snow covers.

Figure S16 shows the measurements and interpolated data of global radiation, relative humidity, temperature and snow cover status for randomly selected hours in 2009.

*2. Potential wind speed (U*_p_*) and wind direction (ddp)*

Interpolation of wind speed data is based on [5], i.e. a standardized (potential) wind speed (*U*_p_) should be interpolated instead of a local wind speed (*U*). Potential wind speed refers to a standardized measurement at a height of 10 m and a site with a roughness length of 3 cm.

We downloaded the potential wind speed (*U*_p_) data, including directions (ddp), from the KNMI database [6, 7] available for 27 measurement stations. Interpolation was performed by means of the IDW method at a 2.5 km raster (*krige::idw( maxdist* *= 150000, idp= 2)*.

For wind direction the same interpolation method was applied. Therefore, the x and y vectors of the potential wind speed were spatially interpolated individually. By means of the interpolated vectors the interpolated wind direction was calculated.

Figure S17 shows the wind speed and wind direction measurements and interpolated data for a randomly selected hour in 2009.

*3. Precipitation amounts (ppA) and duration (ppD)*

Precipitation amounts and precipitation duration was derived from precipitation radar data that were calibrated and made available by the KNMI at HDF5 format [8–11]. We transformed the coordinates of the suspected farms to the precipitation radar projection (proj4-string) “+proj=stere +lat_0=90 +lon_0=0 +lat_ts=60 +a=6378.14 +b=6356.75 +x_0=0 y_0=0”.

The temporal resolution is at five-minute level and the spatial resolution is 1 km. We determined the amount and duration of precipitation per hour, and the average shower length per day (required by the OPS model) using the R-packages *rgdal* and *rhdf5*.

Figure S18 shows a precipitation radar image with the precipitation intensity at several locations in the Netherlands.

**References**

1. **Hourly meteorological data in the Netherlands** [http://www.knmi.nl/klimatologie/uurgegevens/]

2. **Deltares OPeNDAP Meteorological measurement data** [http://opendap.deltares.nl/opendap/knmi/uurgeg/contents.html]

3. Sluiter R: *Interpolation Methods for Climate Data; Literature Review*. De Bilt, The Netherlands: Royal Netherlands Meteorological Institute; 2009.

4. Sluiter R: *Interpolation Methods for the Climate Atlas*. De Bilt, The Netherlands: Royal Netherlands Meteorological Institute (KNMI); 2012(July).

5. Stepek A, Wijnant IL: *Interpolating Wind Speed Normals from the Sparse Dutch Network to a High Resolution Grid Using Local Roughness from Land Use Maps*. De Bilt, The Netherlands: Royal Netherlands Meteorological Insitute; 2011.

6. **Potential wind in the Netherlands** [http://www.knmi.nl/klimatologie/onderzoeksgegevens/potentiele_wind-sigma/ ]

7. **Deltares OPeNDAP Potential wind speed data** [http://opendap.deltares.nl/opendap/knmi/potwind/contents.html]

8. **Product description of the KNMI precipitation radar** [http://adaguc.knmi.nl/contents/datasets/productdescriptions/W_ADAGUC_Product_description_RADNL_OPER_R___25PCPRR_L3.html]

9. Overeem A, Holleman I, Buishand A: **Derivation of a 10-Year Radar-Based Climatology of Rainfall**. *J Appl Meteorol Climatol* 2009, **48**:1448–1463.

10. Overeem A, Buishand TA, Holleman I: **Extreme rainfall analysis and estimation of depth-duration-frequency curves using weather radar**. *Water Resour Res* 2009, **45**.

11. Overeem A, Buishand A: *Statistiek van Extreme Gebiedsneerslag in Nederland*. De Bilt, The Netherlands: Royal Netherlands Meteorological Institute (KNMI); 2012.
