# Supplementary figures and images for: Improved correlation of human Q fever incidence to modelled C. burnetii concentrations by means of an atmospheric dispersion model
Source: Int J Health Geogr. 2015 Apr 1;14:14. doi: 10.1186/s12942-015-0003-y (PMC4440286; doi:10.1186/s12942-015-0003-y)

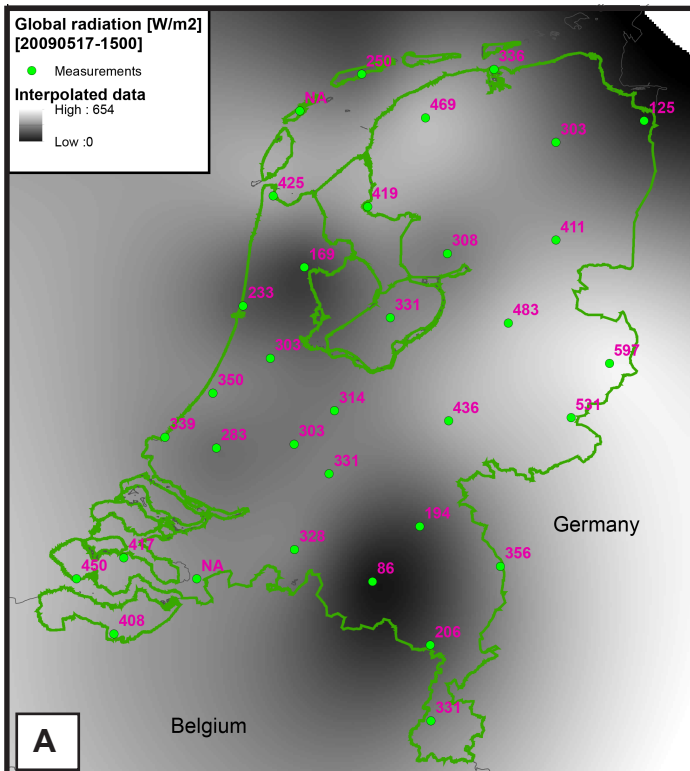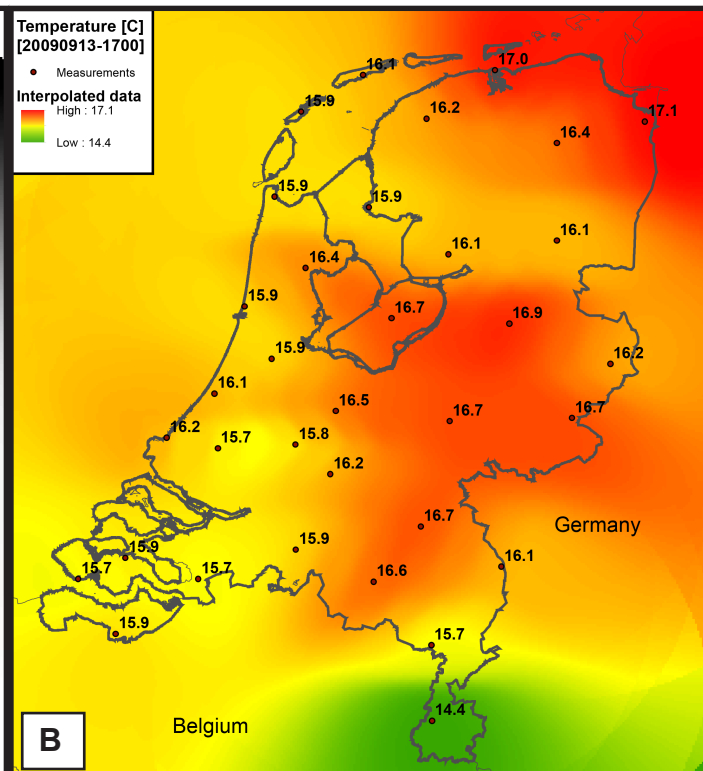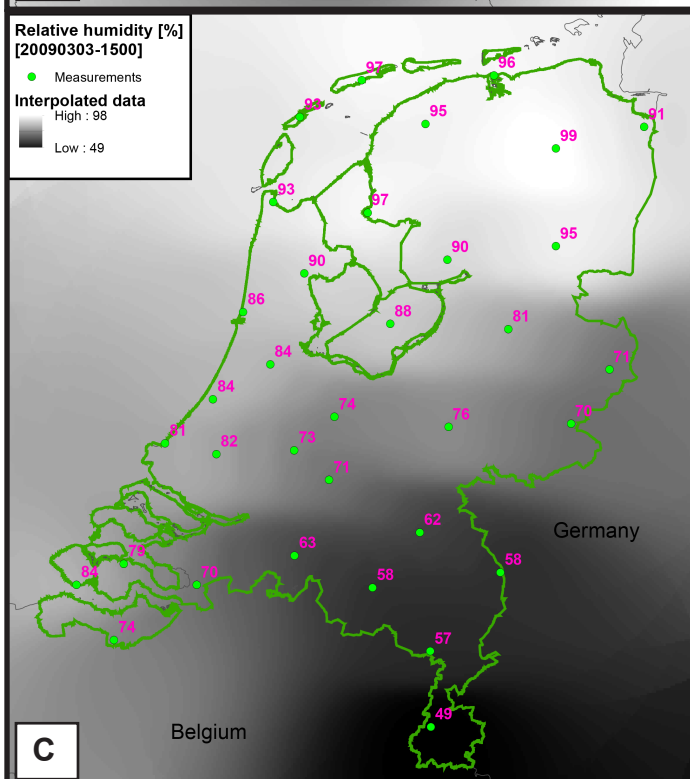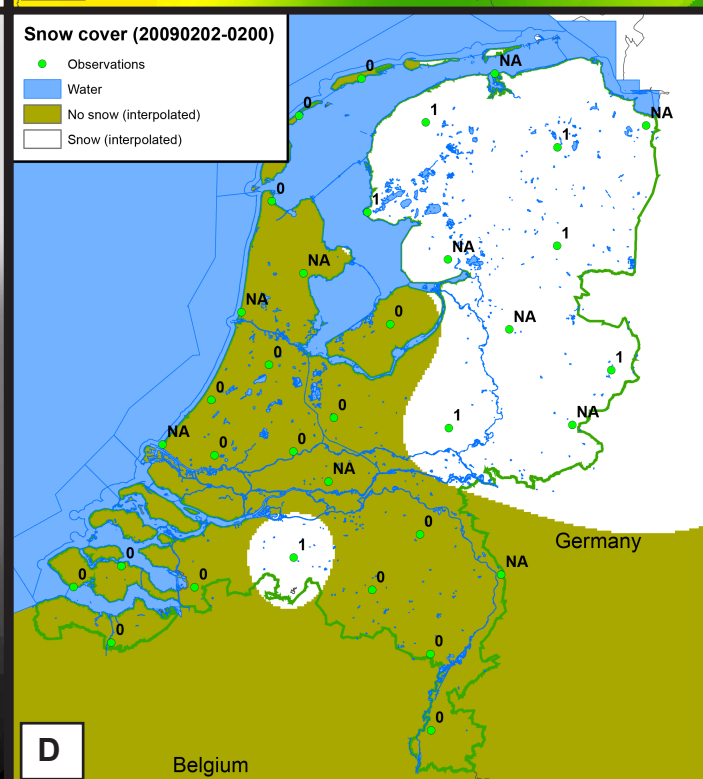

Supplement: Additional file 2: Figure S16. — Interpolation maps (1). Measurements and interpolated data of global radiation, relative humidity, temperature and snow cover status for randomly selected hours in 2009. [file 12942_2015_3_MOESM2_ESM.pdf]

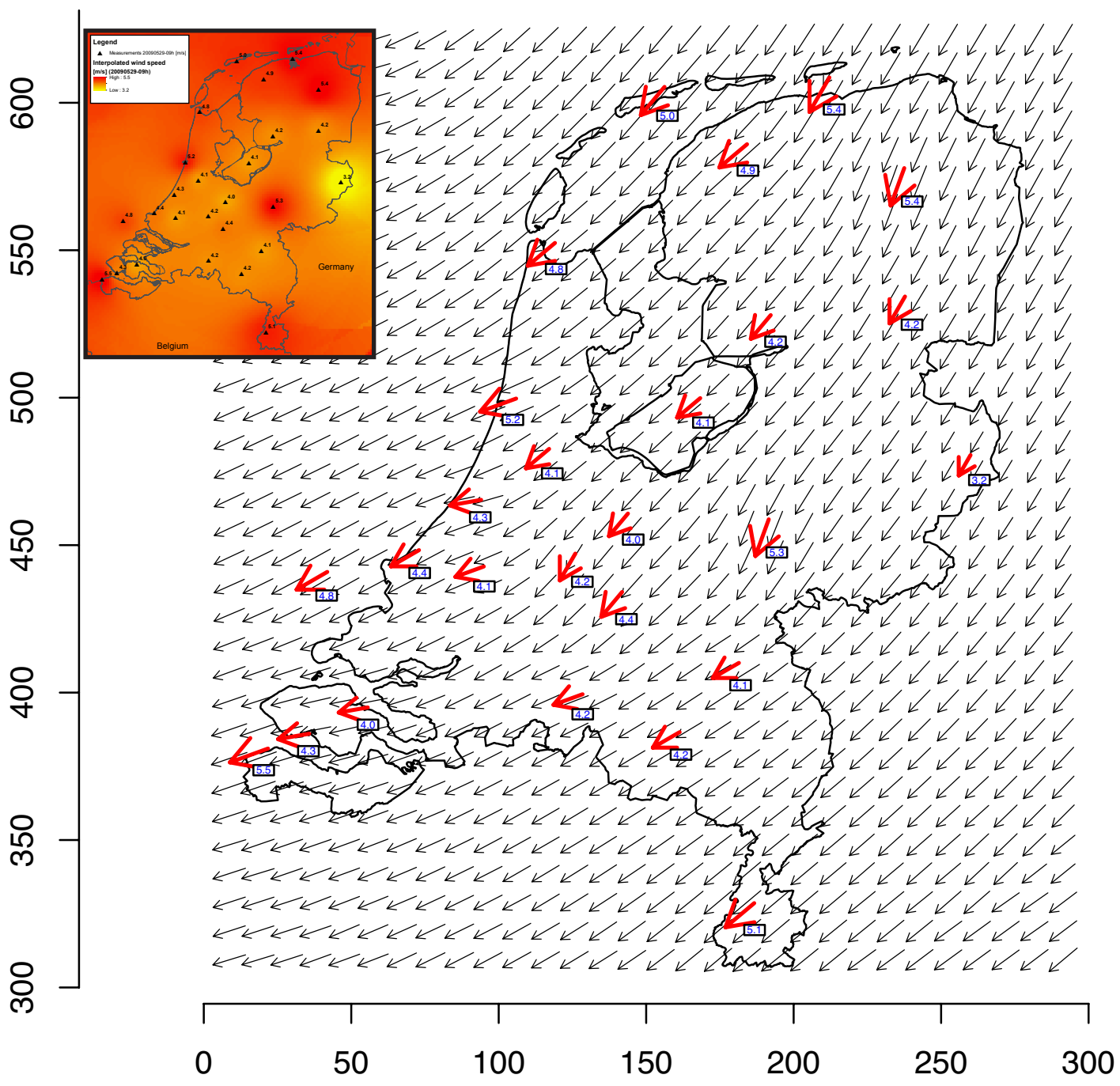

Supplement: Additional file 3: Figure S17. — Interpolation maps (2). Measured and interpolated wind speed and wind direction for a randomly selected hour in 2009. [file 12942_2015_3_MOESM3_ESM.pdf]

### Legend

▲ pplnt at farms [mm/h]

Radar Image 20090526-0200

[mm/h]

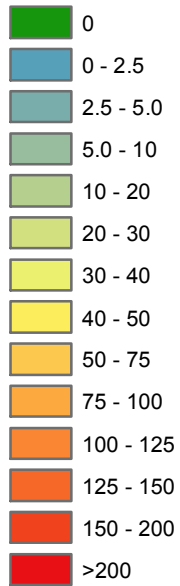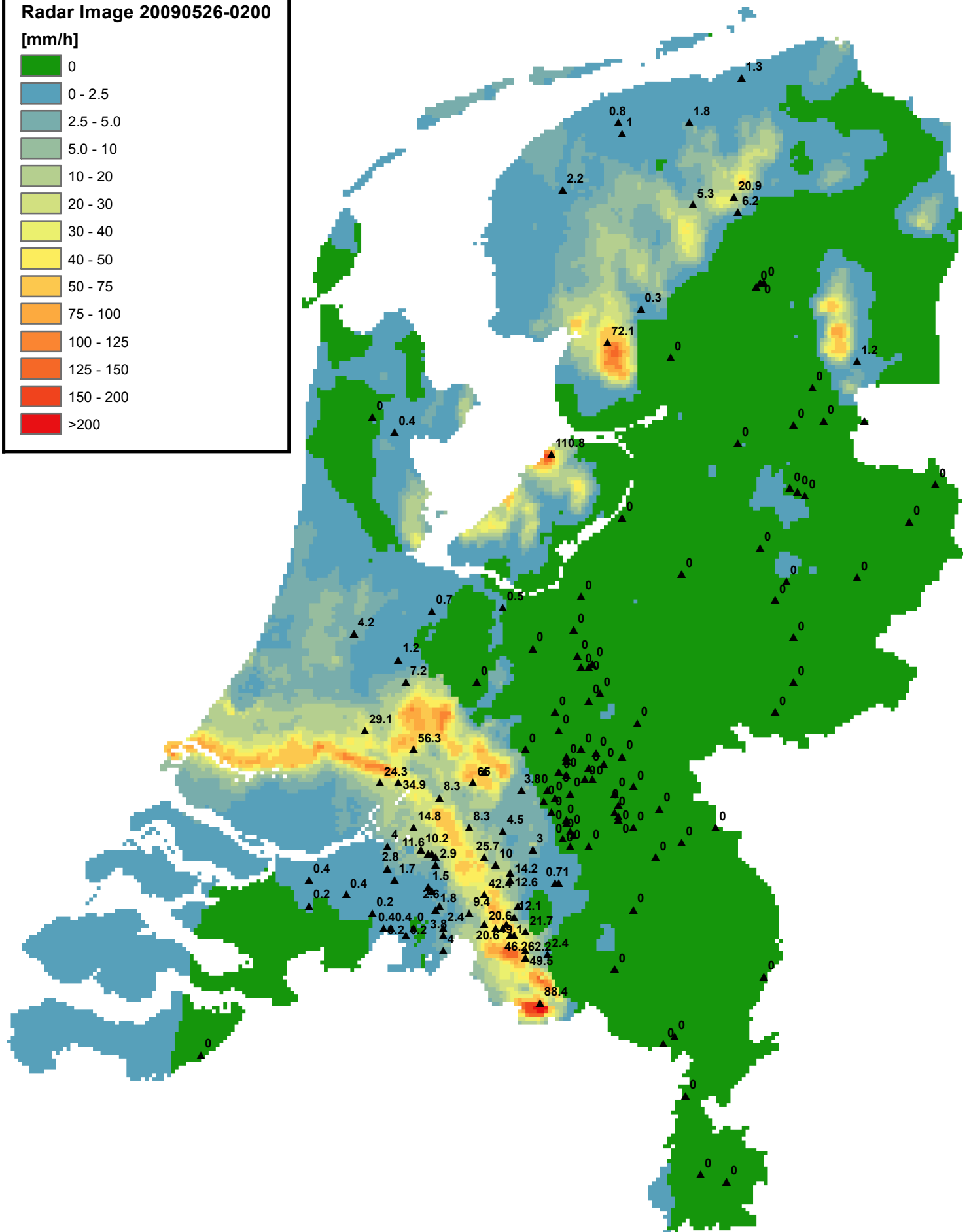

Supplement: Additional file 4: Figure S18. — Precipitation radar image. A semi-random selected precipitation radar image with the converted precipitation intensity at several locations in the Netherlands. [file 12942_2015_3_MOESM4_ESM.pdf]

## area A

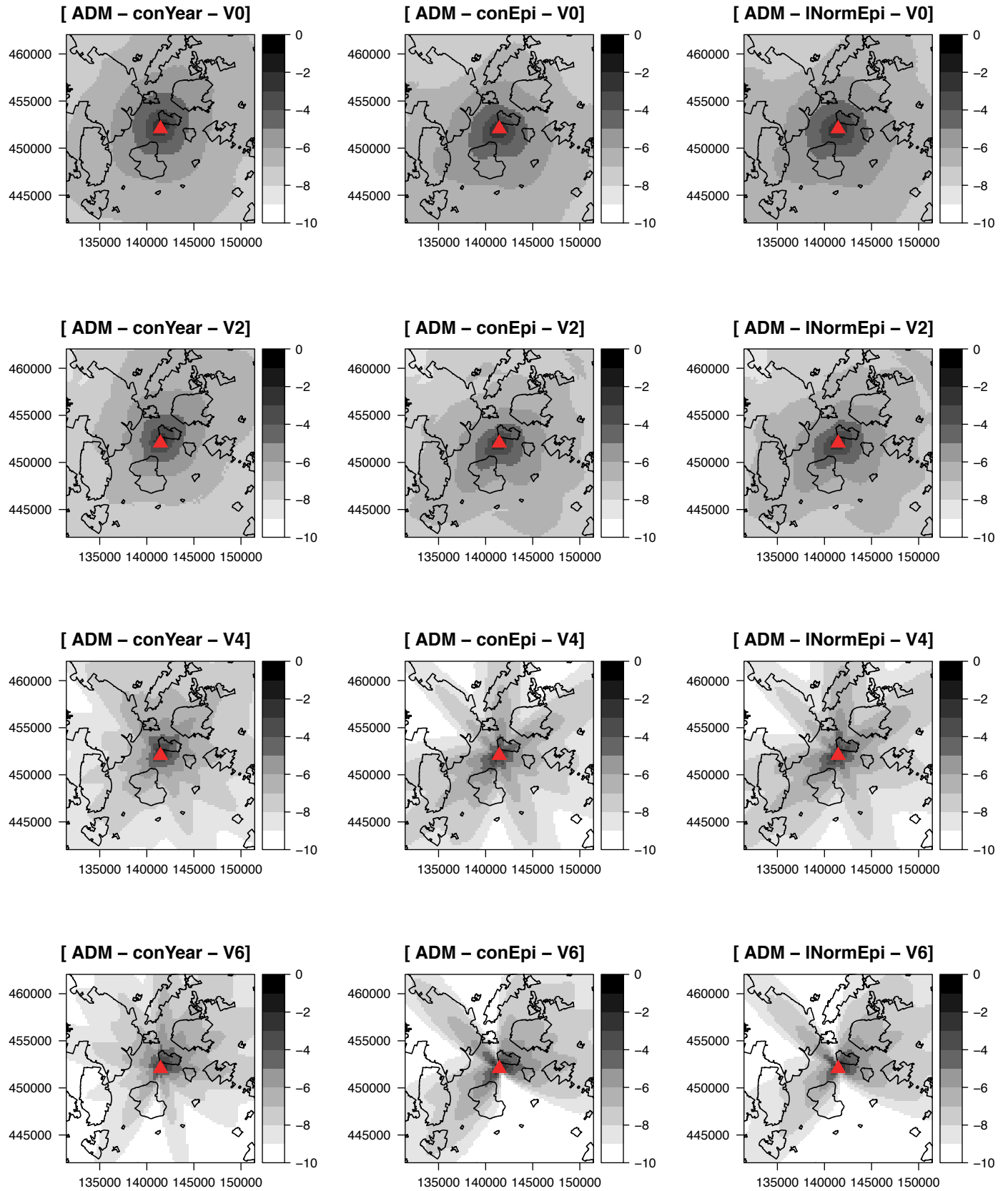

Supplement: Additional file 5: Figure S1. — Concentration maps (area A). Log-transformed ADM concentration maps (relative to the maximum concentration in the grid) with emission profiles conYear, conEpi, and lNormEpi, and threshold wind speed profiles V0, V2, V4 and V6 (area A). [file 12942_2015_3_MOESM5_ESM.pdf]

## area B

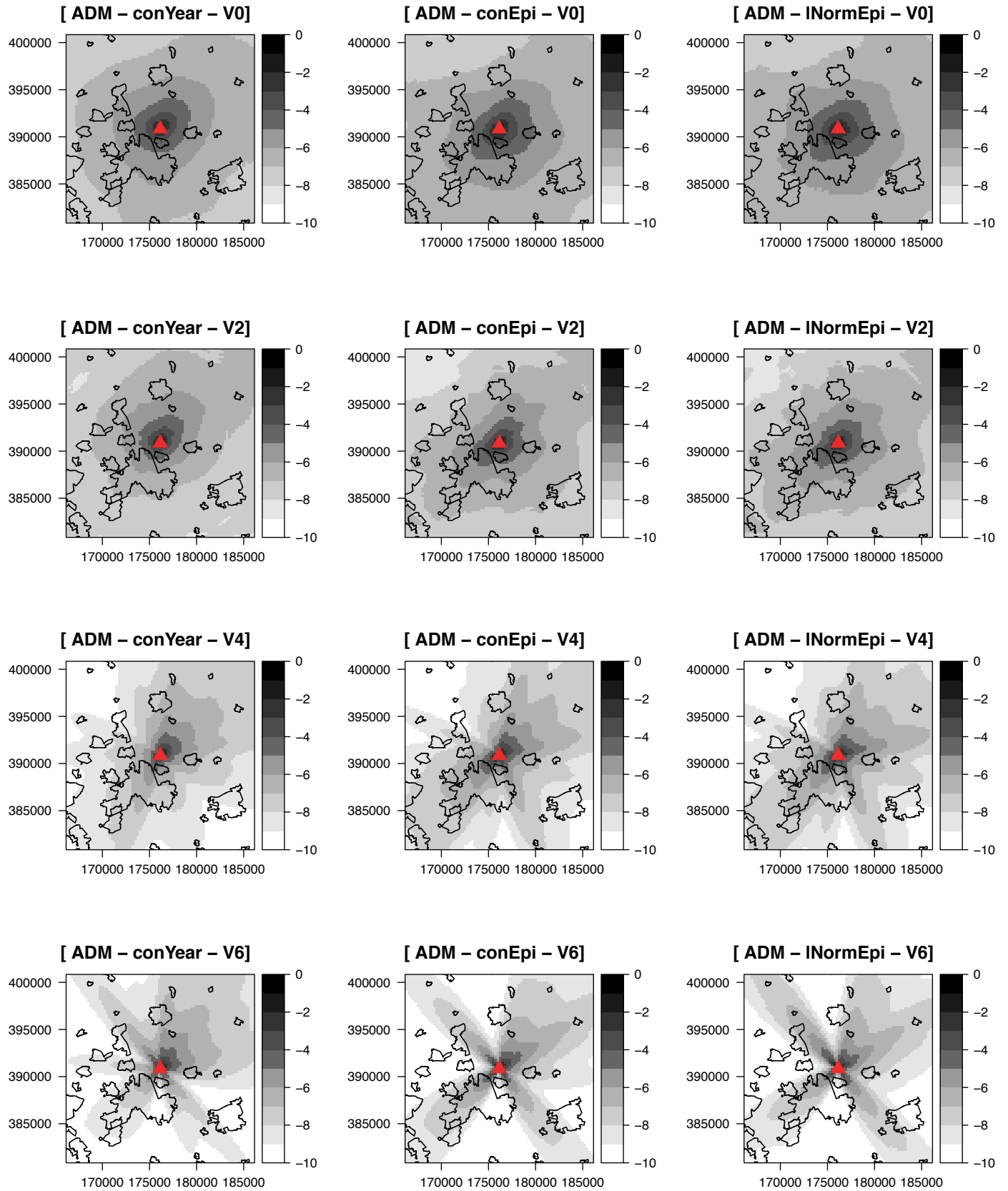

Supplement: Additional file 6: Figure S2. — Concentration maps (area B). Log-transformed ADM concentration maps (relative to the maximum concentration in the grid) with emission profiles conYear, conEpi, and lNormEpi, and threshold wind speed profiles V0, V2, V4 and V6 (area B). [file 12942_2015_3_MOESM6_ESM.pdf]

**area C**

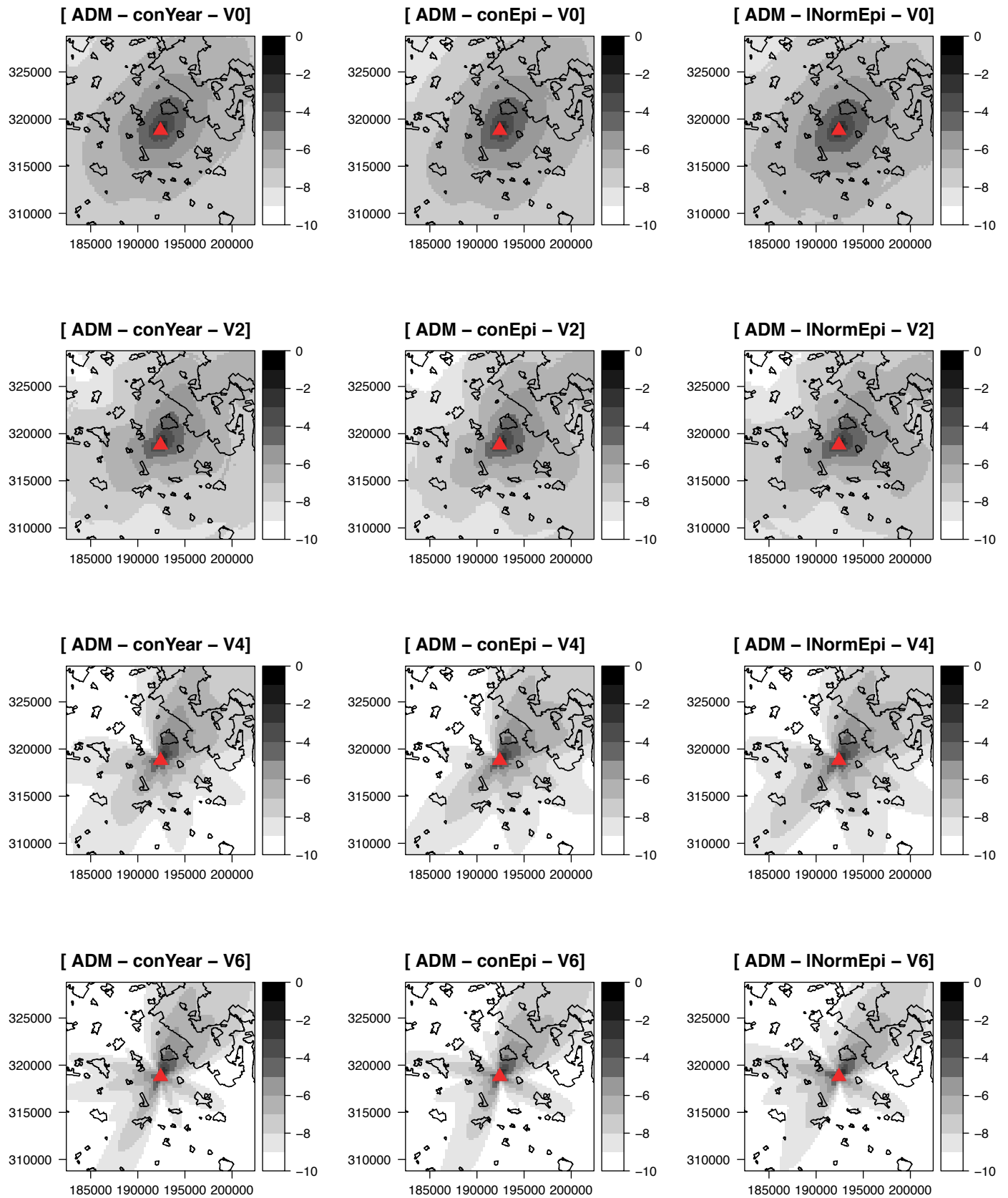

Supplement: Additional file 7: Figure S3. — Concentration maps (area C). Log-transformed ADM concentration maps (relative to the maximum concentration in the grid) with emission profiles conYear, conEpi, and lNormEpi, and threshold wind speed profiles V0, V2, V4 and V6 (area C). [file 12942_2015_3_MOESM7_ESM.pdf]

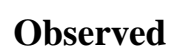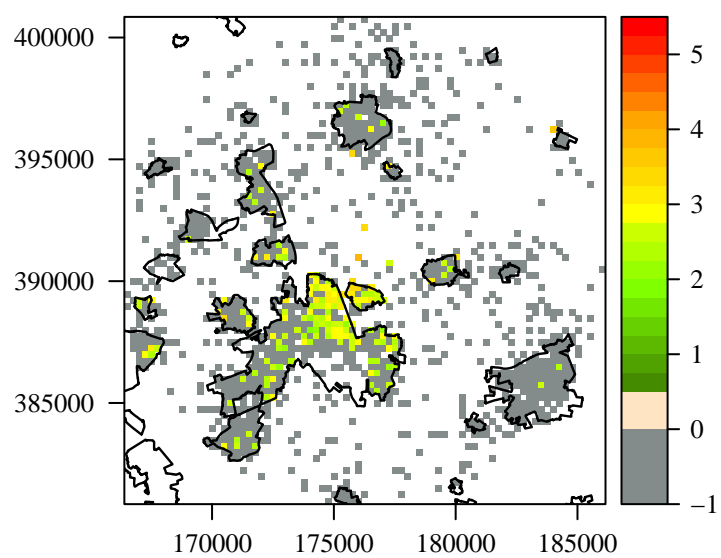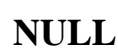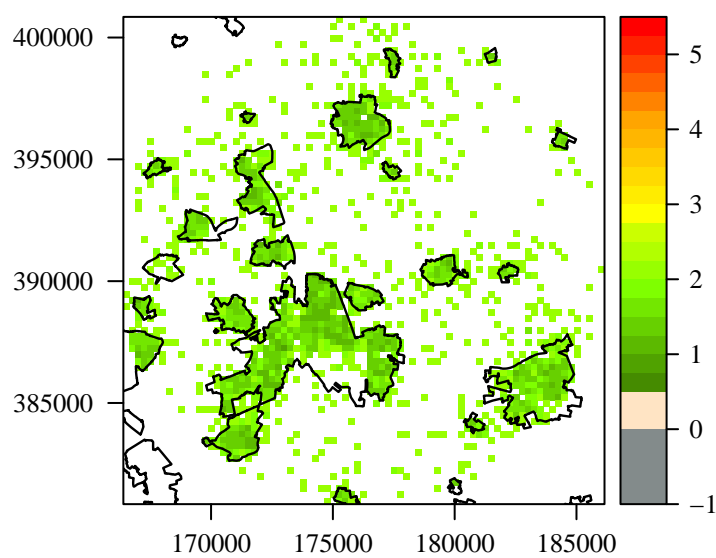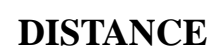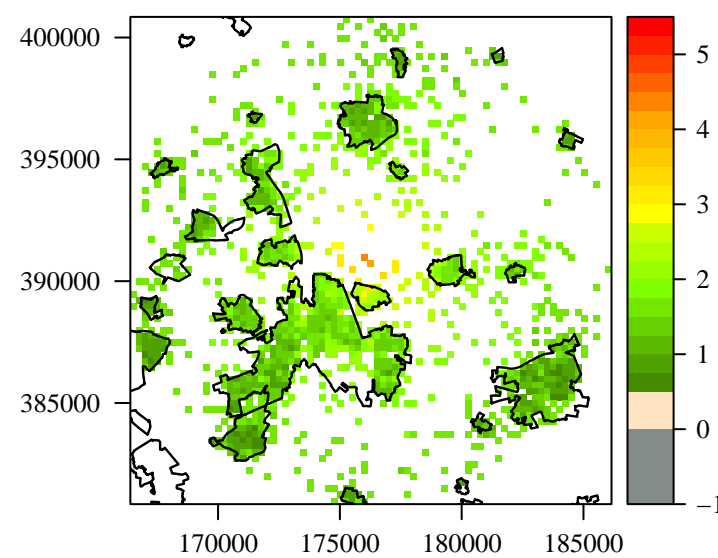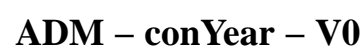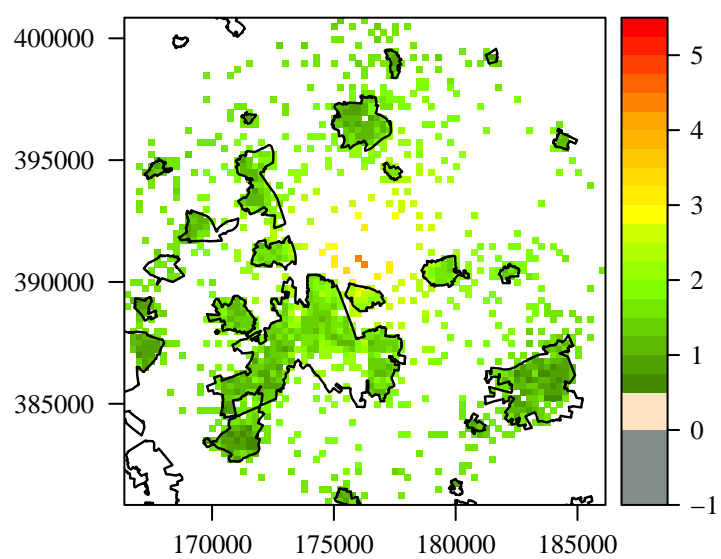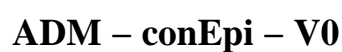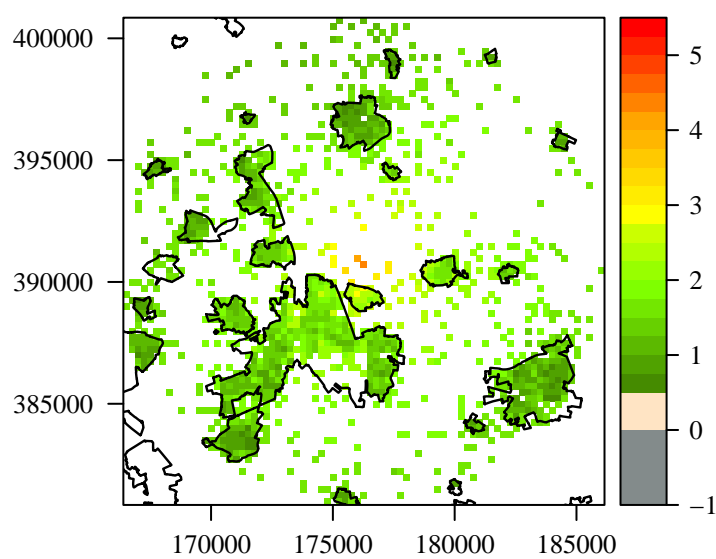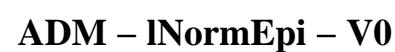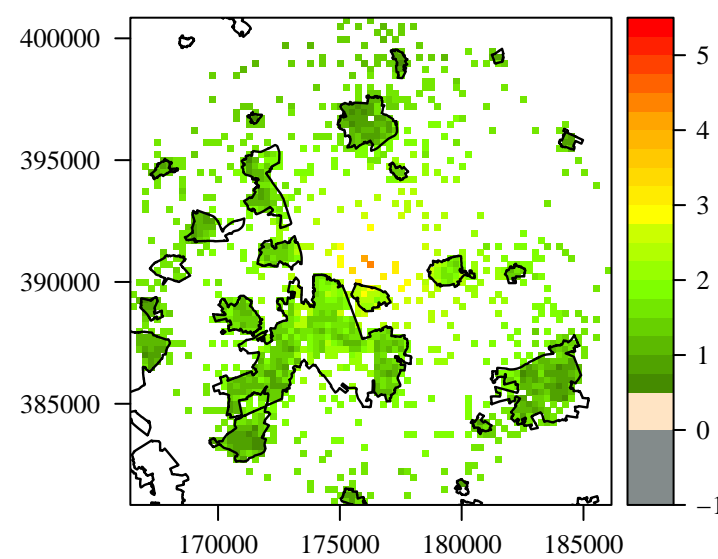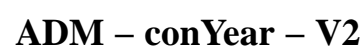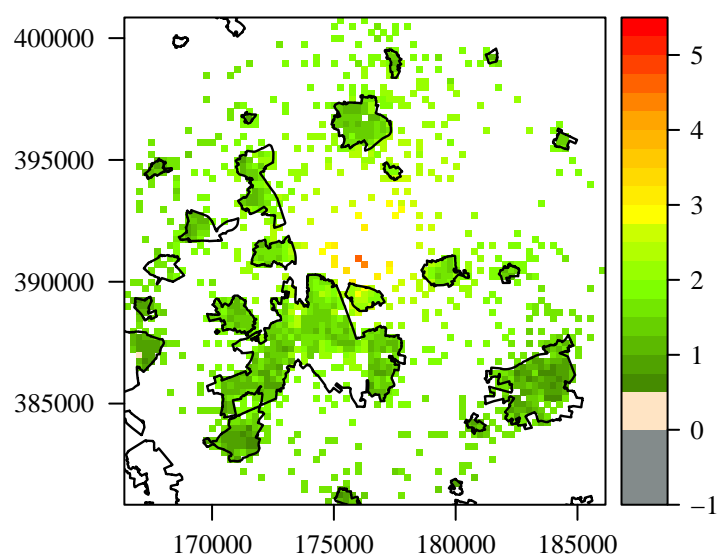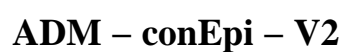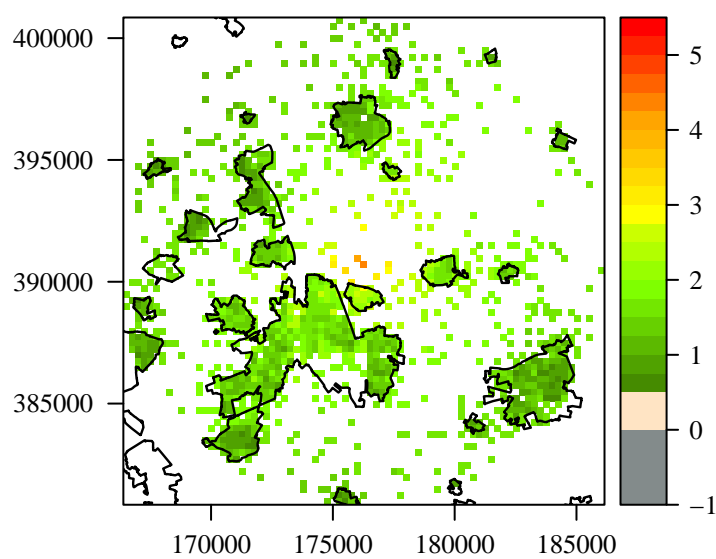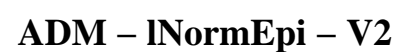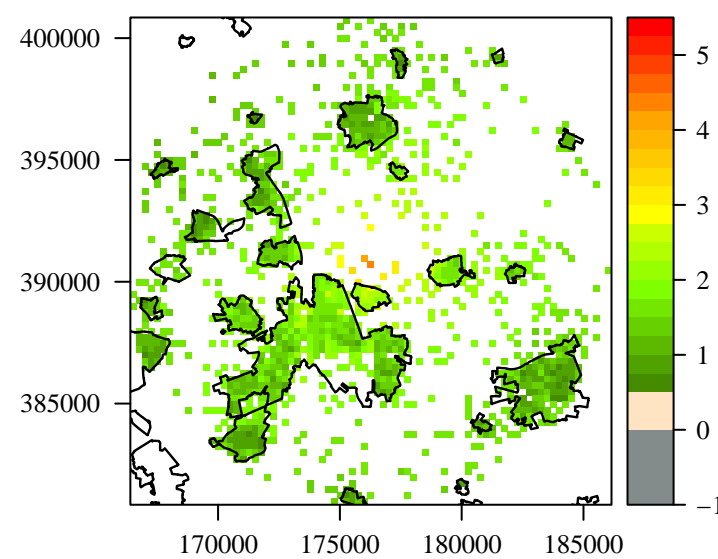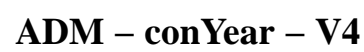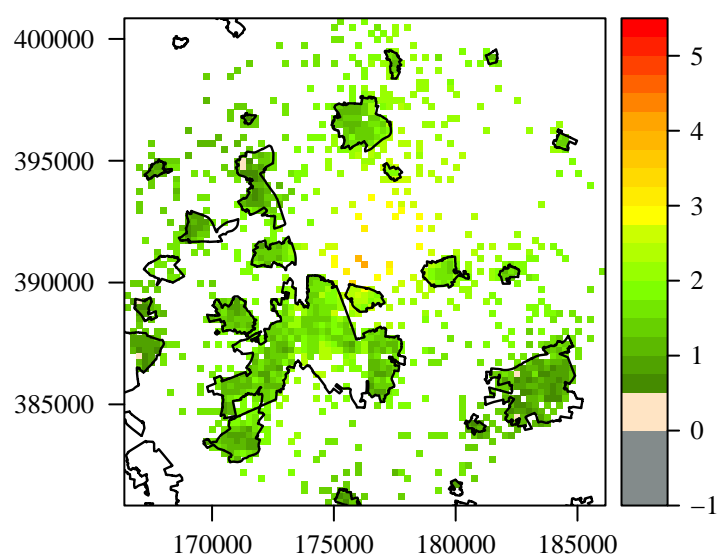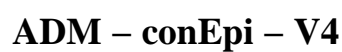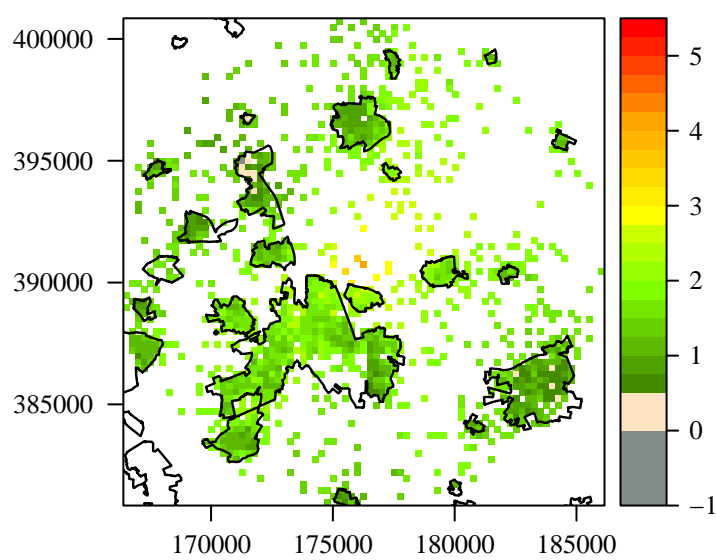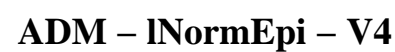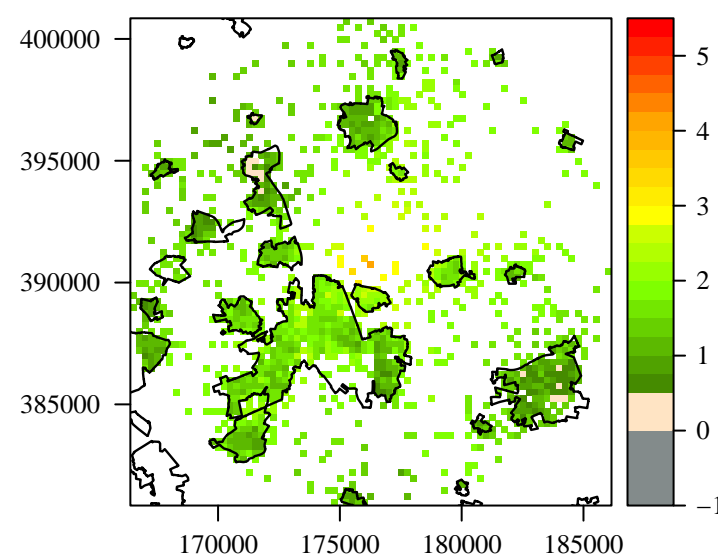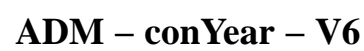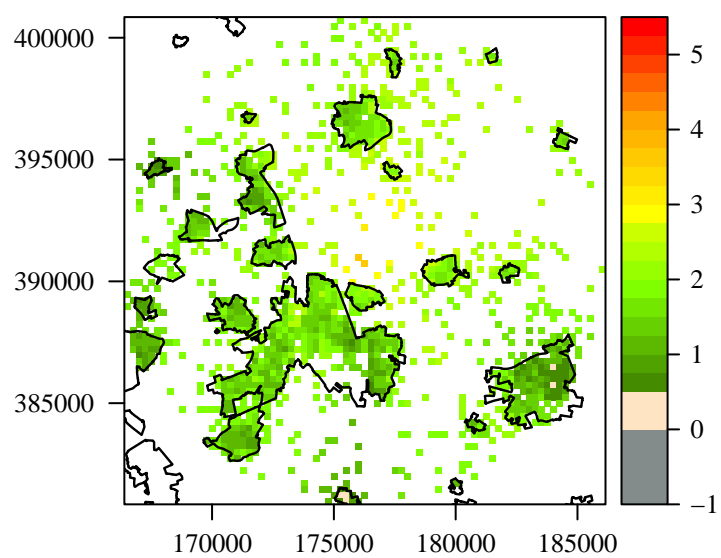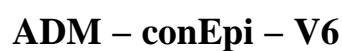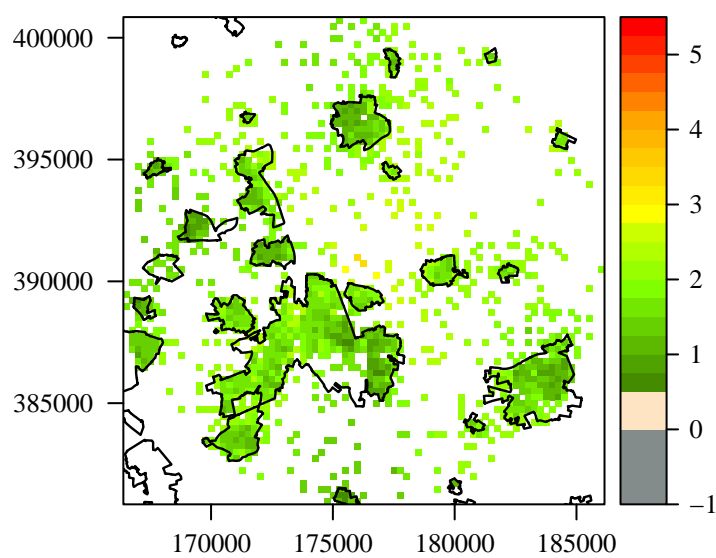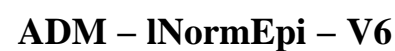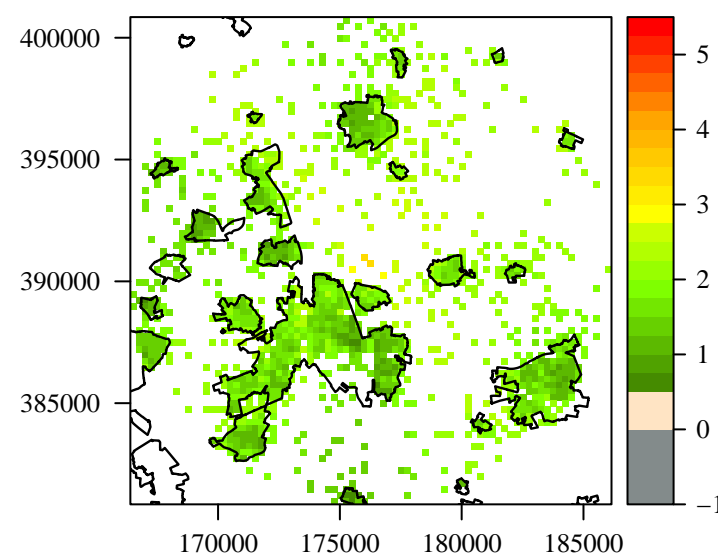

Supplement: Additional file 17: Figure S13. — Geographical observed and predicted incidence map (area A, 10 km). Area A, selection radius 10 km: Geographical observed and predicted incidence rates per 100,000 inhabitants aggregated to a raster at the 250 m level (log10-scale). Grey pixels represent incidence rates of 0. [file 12942_2015_3_MOESM17_ESM.pdf]
