# Supplementary material for: Improved correlation of human Q fever incidence to modelled C. burnetii concentrations by means of an atmospheric dispersion model
Source: Int J Health Geogr. 2015 Apr 1;14:14. doi: 10.1186/s12942-015-0003-y (PMC4440286; doi:10.1186/s12942-015-0003-y)
Supplement: Additional file 18: Figure S14. — Geographical observed and predicted incidence map (area B, 10 km). Area B, selection radius 10 km: Geographical observed and predicted incidence rates per 100,000 inhabitants aggregated to a raster at the 250 m level (log10-scale). Grey pixels represent incidence rates of 0. [file 12942_2015_3_MOESM18_ESM.pdf]

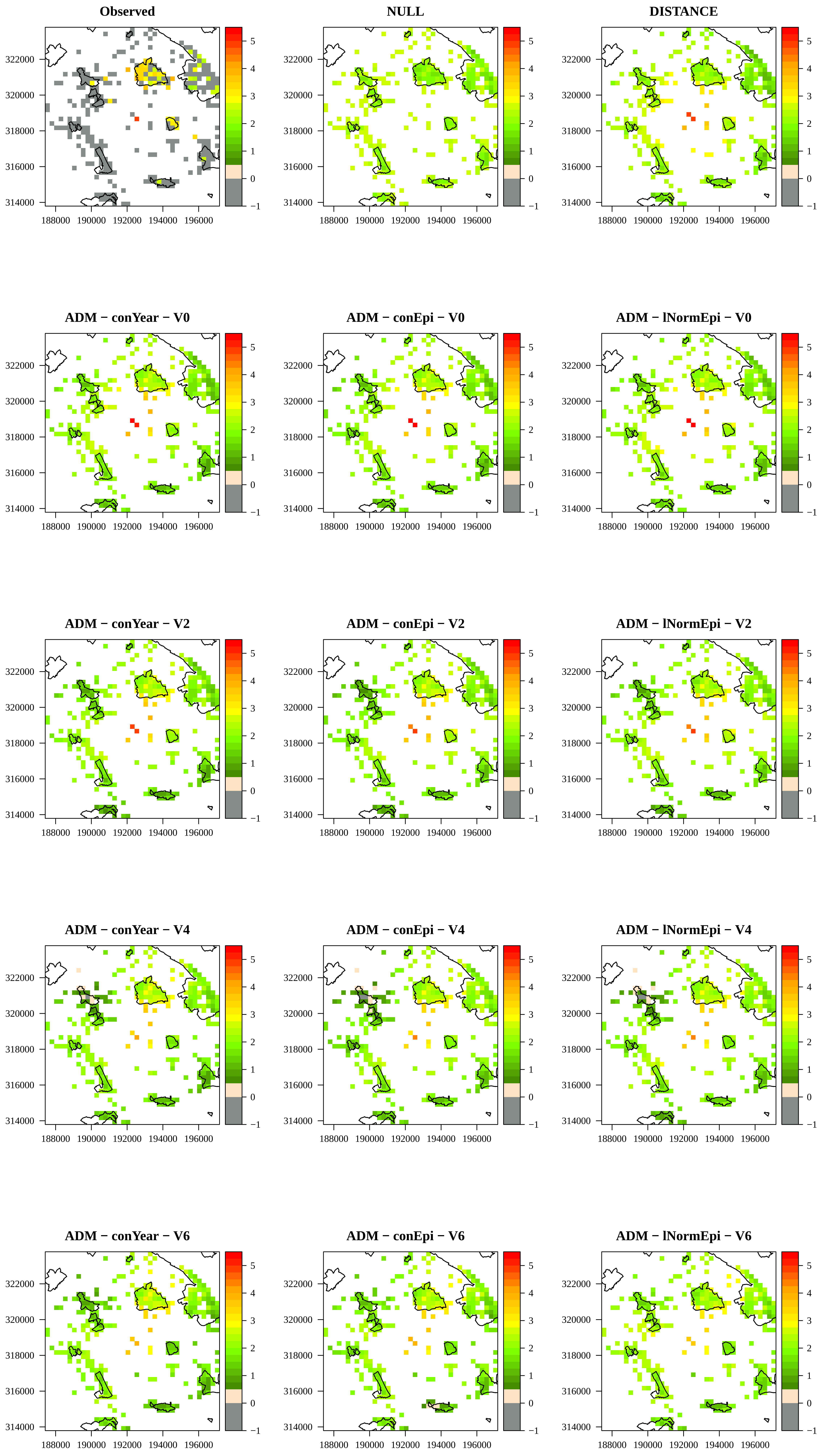
Observed
NULL
DISTANCE
ADM − conYear − V0
ADM − conEpi − V0
ADM − lNormEpi − V0
ADM − conYear − V2
ADM − conEpi − V2
ADM − lNormEpi − V2
ADM − conYear − V4
ADM − conEpi − V4
ADM − lNormEpi − V4
ADM − conYear − V6
ADM − conEpi − V6
ADM − lNormEpi − V6
322000 320000 318000 316000 314000
188000 190000 192000 194000 196000
5 4 3 2 1 0 −1
