# Supplementary material for: Improved correlation of human Q fever incidence to modelled C. burnetii concentrations by means of an atmospheric dispersion model
Source: Int J Health Geogr. 2015 Apr 1;14:14. doi: 10.1186/s12942-015-0003-y (PMC4440286; doi:10.1186/s12942-015-0003-y)
Supplement: Additional file 19: Figure S15. — Geographical observed and predicted incidence map (area C, 10 km). Area C, selection radius 10 km: Geographical observed and predicted incidence rates per 100,000 inhabitants aggregated to a raster at the 250 m level (log10-scale). Grey pixels represent incidence rates of 0. [file 12942_2015_3_MOESM19_ESM.pdf]

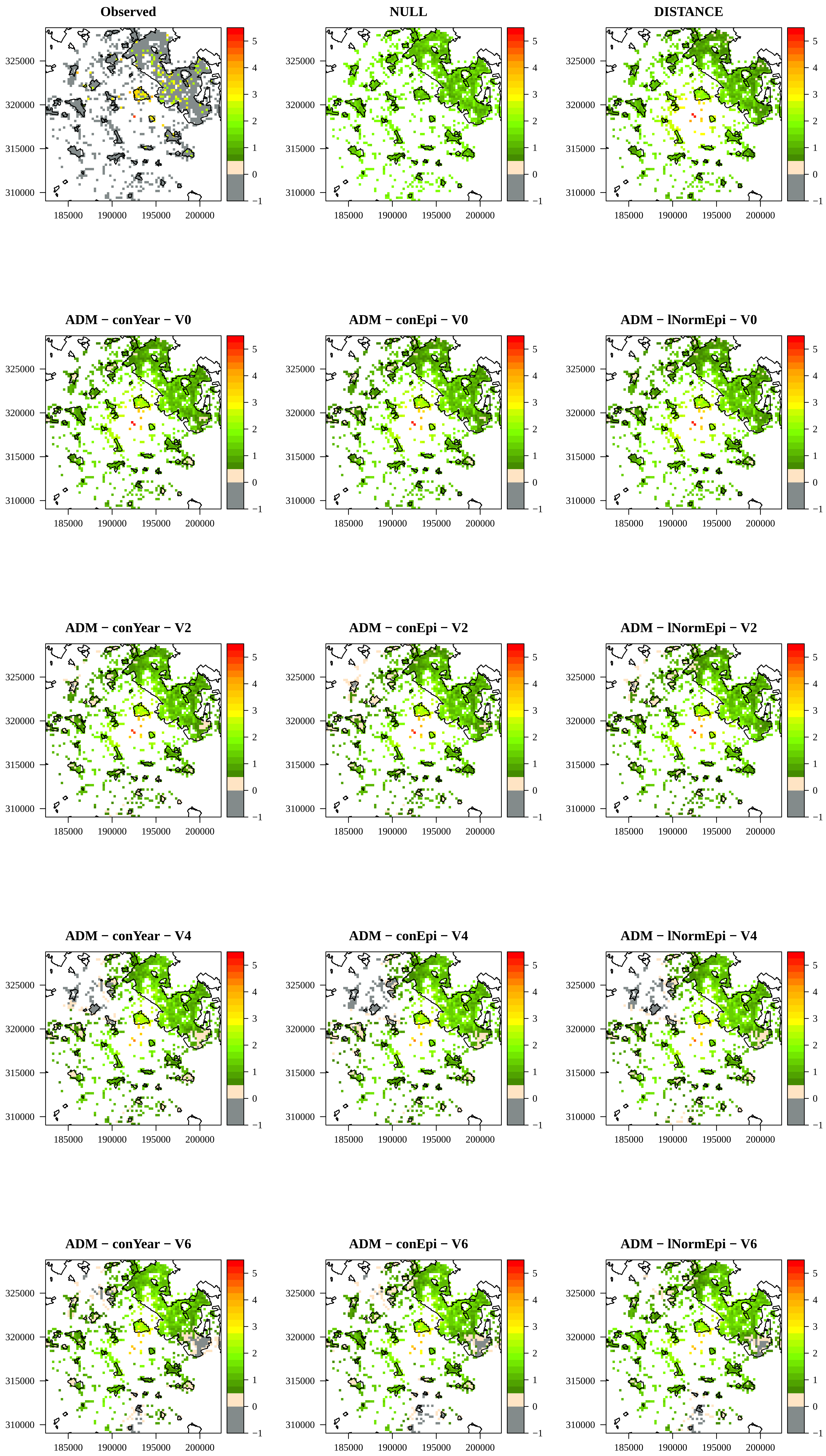
Observed
NULL
DISTANCE
ADM − conYear − V0
ADM − conEpi − V0
ADM − lNormEpi − V0
ADM − conYear − V2
ADM − conEpi − V2
ADM − lNormEpi − V2
ADM − conYear − V4
ADM − conEpi − V4
ADM − lNormEpi − V4
ADM − conYear − V6
ADM − conEpi − V6
ADM − lNormEpi − V6
310000
315000
320000
325000
185000
190000
195000
200000
−1
0
1
2
3
4
5
