# Supplementary material for: Improved correlation of human Q fever incidence to modelled C. burnetii concentrations by means of an atmospheric dispersion model
Source: Int J Health Geogr. 2015 Apr 1;14:14. doi: 10.1186/s12942-015-0003-y (PMC4440286; doi:10.1186/s12942-015-0003-y)
Supplement: Additional file 10: Figure S6. — Predicted versus observed incidence rates (area C, 5 km). Area C, selection radius 5 km: Predicted (y) versus observed (x) incidence rates per PC6 for the NULL, DISTANCE and ADM models. The solid line displays the 1×1 curve. PC6’s with no observed cases are not included. [file 12942_2015_3_MOESM10_ESM.pdf]

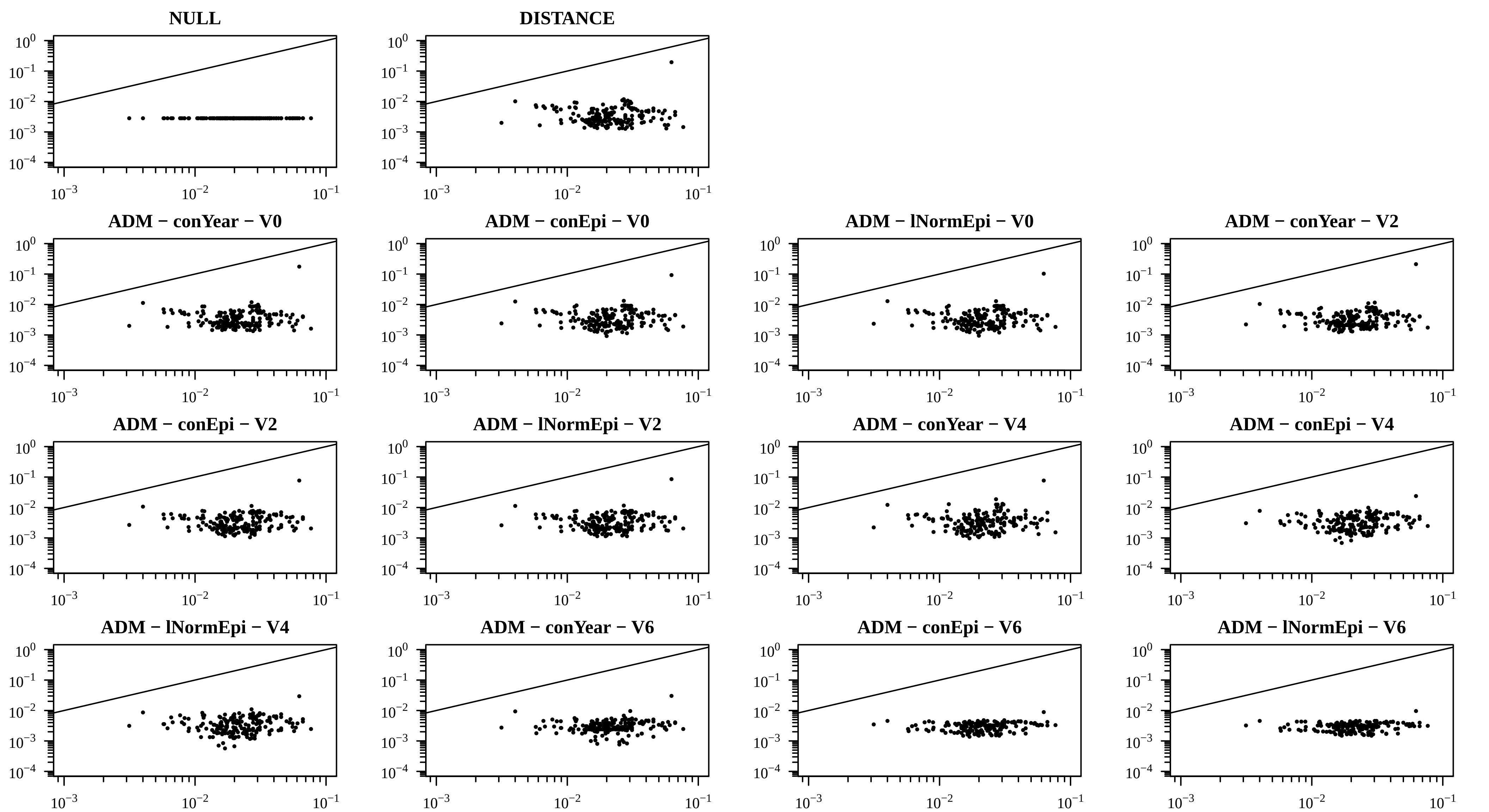
NULL
DISTANCE
ADM − conYear − V0
ADM − conEpi − V0
ADM − lNormEpi − V0
ADM − conYear − V2
ADM − conEpi − V2
ADM − lNormEpi − V2
ADM − conYear − V4
ADM − conEpi − V4
ADM − lNormEpi − V4
ADM − conYear − V6
ADM − conEpi − V6
ADM − lNormEpi − V6
10^{0}
10^{-1}
10^{-2}
10^{-3}
10^{-4}
10^{-3}
10^{-2}
10^{-1}
